# Covalent‐Allosteric Inhibitors to Achieve Akt Isoform‐Selectivity

**DOI:** 10.1002/anie.201909857

**Published:** 2019-11-08

**Authors:** Lena Quambusch, Ina Landel, Laura Depta, Jörn Weisner, Niklas Uhlenbrock, Matthias P. Müller, Franziska Glanemann, Kristina Althoff, Jens T. Siveke, Daniel Rauh

**Affiliations:** ^1^ Faculty of Chemistry and Chemical Biology TU Dortmund University and Drug Discovery Hub Dortmund (DDHD) Zentrum für Integrierte Wirkstoffforschung (ZIW) Otto-Hahn-Strasse 4a 44227 Dortmund Germany; ^2^ Institute of Developmental Cancer Therapeutics West German Cancer Center, University Hospital Essen Essen Germany; ^3^ Division of Solid Tumor Translational Oncology German Cancer Consortium (DKTK, partner site Essen) and German Research Center (DKFZ) Heidelberg Germany

**Keywords:** Akt isoforms, allosteric sites, cancer, covalent inhibitors, isoform selectivity

## Abstract

Isoforms of protein kinase Akt are involved in essential processes including cell proliferation, survival, and metabolism. However, their individual roles in health and disease have not been thoroughly evaluated. Thus, there is an urgent need for perturbation studies, preferably mediated by highly selective bioactive small molecules. Herein, we present a structure‐guided approach for the design of structurally diverse and pharmacologically beneficial covalent‐allosteric modifiers, which enabled an investigation of the isoform‐specific preferences and the important residues within the allosteric site of the different isoforms. The biochemical, cellular, and structural evaluations revealed interactions responsible for the selective binding profiles. The isoform‐selective covalent‐allosteric Akt inhibitors that emerged from this approach showed a conclusive structure–activity relationship and broke ground in the development of selective probes to delineate the isoform‐specific functions of Akt kinases.

## Graphical abstract

**Akt now**! A structure and homology model guided approach for the design of diverse and pharmacologically beneficial covalent‐allosteric modifiers for protein kinase Akt is presented. The isoform‐selective Akt inhibitors show a conclusive structure–activity relationship and break ground for further development of selective probes for the dissection of Akt isoform‐specific functions.

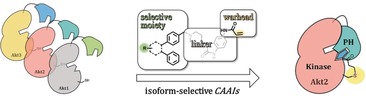

The central role of protein kinase Akt in proliferative signaling pathways renders it an essential target for therapeutic applications. Dysregulation correlates with different diseases such as cancer, diabetes, cardiovascular or neurologic malfunctions.^1^ In cancer, over‐activated upstream mediators, as well as loss of function mutations in the tumor suppressor PTEN but not necessarily mutations in Akt, lead to a constitutive activation of Akt enzymes and make them important targets for therapeutic intervention.^2^

In humans, the three isoforms Akt1, Akt2, and Akt3 (also termed protein kinase B PKB‐α, ‐β, and ‐γ, respectively) are known. The isoforms show a high sequence homology (identity of 73 %, see the Supporting Information, Figure S1). However, their intracellular localizations and functions differ; Akt1 is ubiquitously localized in the cytosol and partially at the plasma membrane, Akt2 is concentrated in mitochondria and has been reported to associate with mitochondrial hexokinase, and Akt3 is co‐localized with the nucleus.^3^ Phenotypic knock‐out studies in mice have shown that the diversity within the Akt‐mediated pathways relies on specific isoform functions. Akt1 is related to proliferation and antiapoptotic behavior.^4^ Akt2 deletion leads to hyperglycemia, a type‐2 diabetic phenotype, and the impairment of glucose uptake.^5^ Absence of Akt3 results in neuronal malfunction and altered fatty acid metabolism.^6^ Recent knock‐down studies underlined the opposing roles of Akt1 and Akt2 in different cancer types. Within the context of aggressive forms of breast cancer, Akt2 seems to be responsible for metastasis and invasiveness in advanced stages, suggesting a selective inhibition of Akt2 as a favorable therapeutic strategy.^7^ A different behavior was reported for lung cancer, in which Akt1 functions as a tumor initiator whereas Akt2 had suppressive characteristics, suggesting an Akt1 selective strategy.^8^

It is noteworthy that all results on isoform‐specific functions within this intricate signaling network are based on knock‐down studies. A thorough investigation with chemical tools would help to further elucidate this complex network and the interplay through minimally invasive perturbation studies.^9^ Such a strategy necessitates bioactive ligands with a defined selectivity profile for each isoform. The gain of selectivity for certain highly similar isoforms of a protein is a central issue in drug and probe development, and challenging examples for which isoform‐selective molecules were found include the recently observed GPCR‐ as well as HDAC‐selective inhibitors.^10^ These concerns account for enzyme selectivity within the highly homologous kinase family as well as targeting certain disease‐causing mutants while sparing the wildtype protein.^11^ In the case of Akt, the known clinically evaluated and well‐described ATP‐competitive ligands are pan‐inhibitors and they lack isoform‐selectivity.^12^ A promising novel class of inhibitors to overcome selectivity issues was introduced recently, which presented allosteric and covalent binding Akt inhibitors (CAAIs).^13^ The prototype of this innovative class of compounds is borussertib, which alkylates one of the two cysteine residues in a unique interdomain pocket between the regulatory PH and kinase domain and irreversibly stabilizes an inactive conformation with a structurally blocked ATP‐binding site (Figure 1 C). Besides their pharmacological benefit of targeting a covalent anchor point, derivatives of borussertib exhibit a slight preference for different isoforms.^14^ Based on these preliminary results, a structure‐guided approach has led to a set of structurally diverse and pharmacologically beneficial covalent modifiers that can be utilized for further investigation of isoform‐specific preferences and isoform‐selective binding residues. Herein, we describe the first set of these isoform‐selective covalent‐allosteric Akt inhibitors that show a conclusive structure–activity relationship (SAR) and break ground for further probe development. We identified molecules that preferably bind to Akt1 as well as molecules that demonstrated a good selectivity profile for Akt2. Furthermore, we solved two full‐length crystal‐structures of Akt1 in complex with this novel class of ligands and validated their selectivity within cellular models in immunostaining experiments.

Owing to the lack of full‐length crystal structures of Akt2 and Akt3, we performed a detailed homology and 2D‐structure‐based modeling for possible identification of isoform‐specific changes within residues forming the interdomain pocket (Figure 1 A,B and Figure S1). The comparison of the sequence to the full‐length Akt1 structure allowed us to evaluate possible structural differences. Most of the sequence is conserved and chemical properties of altered amino acids are similar (e.g., Ser 205 is a threonine residue in Akt2 and Akt3, or Lys 268 is an arginine residue in Akt2). However, some differences surrounding the allosteric binding pocket can be identified; amino acid deletions that presumably affect the size of the pocket occur in the loop 259–273. In Akt2, a deletion next to Arg 268 might disrupt the helical structure and therefore reduce the local rigidity, resulting in a more flexible solvent‐exposed site of the pocket in Akt2 compared to Akt1. Additionally, Val 270 is replaced in Akt3 by an isoleucine residue, which might occupy more space based on an additional carbon, and another deletion right before this residue might further reduce the size of the allosteric pocket. A homology model supported these assumptions, indicating a shortened helical structure around residue 265–270 and an enlarged solvent‐exposed site away from the PH‐domain interface in Akt2 and Akt3 (Figure S2).^15^ Thus, the slight alterations surrounding the allosteric binding pocket might provide hints towards the binding preferences for specific moieties and facilitate a guided drug‐design approach. These insights let us to speculate that a modification strategy of known ligands either towards the solvent‐exposed kinase domain or deeper into the PH‐domain interface might provide isoform selectivity. This proposal guided our design of a focused ligand set based on previously described regioisomeric Akt inhibitors with either a substitution at the 5′ or 6′‐position of the six‐membered ring system (Figure 1 D)^16^

**Figure 1:**
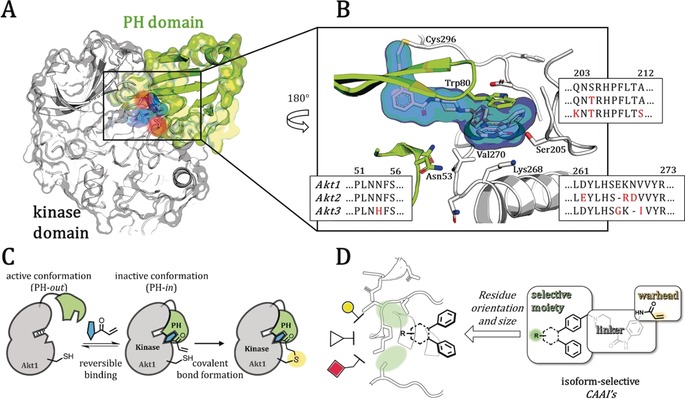
Covalent‐allosteric Akt inhibitors as a beneficial starting point to serve as tools for investigation of isoform selectivity. A) Co‐crystal structure of full‐length Akt1 in complex with a covalent‐allosteric inhibitor (highlighted in blue, PDB: 6HHI) shows covalent binding to one of two conserved cysteines (red) and stabilizes an inactive conformation through interaction with the kinase domain (white) and the PH‐domain (green). B) Detailed view of the interdomain binding pocket in comparison with an Akt‐isoform sequence alignment (with BLAST); alterations of amino acids (red). C) General scheme of the conformational changes upon allosteric ligand binding; first a reversible step takes place followed by the irreversible modification. D) Design of covalent‐allosteric ligands, which contain different moieties and orientations towards the binding pocket part where amino acid alterations in the Akt‐isoforms occur.

For the effective evaluation of the selectivity of the Akt isoforms, we developed a divergent synthesis route with late‐stage diversity‐oriented functionalization to generate a focused set of regioisomeric covalent‐allosteric inhibitors. The synthetic strategy was designed to generate the pyrazinones and also to allow introduction of distinct structural and chemical features within the derivatization step (Scheme 1). A partially convergent synthesis of building blocks **4** and **9** was developed to easily generate the diketone intermediate **10**, which gives access to a variety of cyclization reactions. The synthetic route for diketone intermediate **9** started from acetylene **5**, being transformed into the alkyne **7** through a Sonogashira cross‐coupling reaction. The oxidation with permanganate led to the diketone **8**, followed by a selective bromination at the aliphatic methyl to yield fragment **9**.^17^ In contrast, the synthesis of benzo[*d*]imidazolone **4** started with a selective *ortho*‐nitration to obtain **2**.^18^ A Pd‐catalyzed reduction of the introduced nitro‐group plus a dehalogenation resulted in intermediate **3**. The final fragment **4** was obtained after a regioselective Boc‐protection of the anilinic amine.^19^ A conventional nucleophilic substitution of the building blocks **4** and **9** gave the diketone intermediate **10**. Proceeding with this intermediate, the acid‐mediated cyclization in the presence of a variety of *α*‐aminocarboxamides yielded a diverse set of regioisomeric pyrazinones **11 a**–**c** and **12 a**–**c**.^16^ The deprotection of the Boc‐group followed by attachment of the acrylamide resulted in the final molecules **15 a**–**c** and **16 a**–**c** which were decorated with electrophilic warheads. In total, twelve novel inhibitors were synthesized including the corresponding reversible anilinic derivatives (**13 a**–**c** and **14 a**–**c**).

**Scheme 1:**
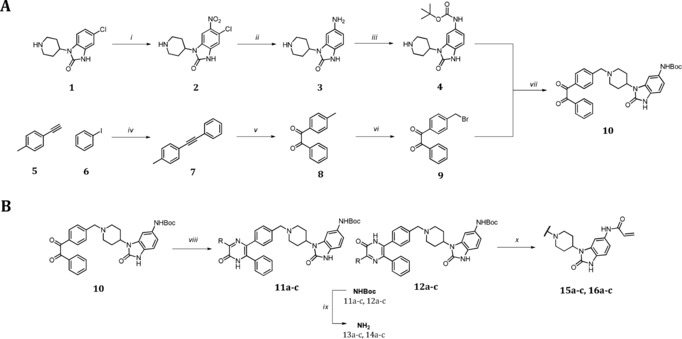
Partial convergent synthesis route with late stage functionalization followed by introduction of the electrophilic warhead. A) 70 % HNO_3_, *o*‐xylene, 60 °C, 1.5 h (i),Pd/C, NH_4_HCOO, MeOH, 80 °C, 16 h (ii), BocO_2_, AcOH/1,4‐dioxane, rt, 16 h (iii), Pd(PPh_3_)_4_, CuI, *i*Pr_2_NH, 60 °C, 10 h (iv), KMnO_4_, NaHCO_3_, MgSO_4_, acetone/H_2_O, rt, 4 h (v), NBS, AIBN, TCM, 90 °C, 30 min (vi). B) Cyclization of regioisomeric pyrazinones: DIPEA, THF, rt, 2 h (vii), aminocarboxamide, EtOH, AcOH (5 %), 80 °C, 1 d, then NaOH_aq_, rt, 12 h (viii), 1 m HCl in 1,4‐dioxane, rt, 12 h (ix), acryloylchloride, DIPEA, THF, 0 °C to rt, 12 h (x).

In order to evaluate the selectivity of the focused ligand set, the inhibitory potency (IC_50_) towards all three full‐length Akt isoforms was determined in a biochemical activity‐based assay (Table 1). Notably, the electrophilic warhead‐modified ligands showed a clear improvement in potency, around a factor of 10–20, compared to the reversible inhibitors. The methyl pyrazinones indicated a good selectivity towards Akt1, especially **15 a** demonstrated a strong loss of activity toward Akt2 and Akt3. Bulky groups, like the isobutyl and the hydroxybenzoyl moiety, were less well tolerated by Akt1, and particularly the 5′‐ derivatives were unfavored compared to the 6′‐position. An opposing effect was observed for Akt2 and Akt3, which seemed to preferentially bind the bulky 5′‐position derivatives and were less tolerant toward the other regioisomer. Strikingly, molecule **16 b** displayed the best selectivity toward Akt2 with a 10‐fold higher affinity in comparison to Akt1, as indicated by the selectivity ratio *α*. Ligand **16 c** was an almost equipotent ligand for Akt1 and Akt3, exhibiting a 5‐fold lower activity toward Akt2 with an overall moderate affinity.

**Table 1:** Biochemical evaluation of regioisomeric covalent‐allosteric Akt inhibitors with the Akt isoforms. 
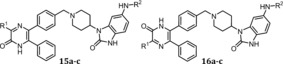

			Akt1^wt^	Akt2^wt^	Akt3^wt^	Selectivity α	
#	R^1^	R^2^	IC_50_ [nm]	IC_50_ [nm]	IC_50_ [nm]	Akt1/Akt2	Akt1/Akt3
capivasertib			1±0.1	5±0.4	8±1	3	4
MK‐2206			10±2	157±45	951±291	10	59
borussertib		‐acryl	1±0.3	56±1	618±24	56	618
**13 a**	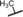	‐H	319±166	>20 000	>20 000	–	–
**15 a**		‐acryl	38±6	1569±302	>20 000	41	–
**14 a**		‐H	3206±1353	11 541±1105	>20 000	4	–
**16 a**		‐acryl	39±5	13 030±1147	>20 000	334	–
**13 b**	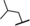	‐H	5415±1695	>20 000	>20 000	–	–
**15 b**		‐acryl	490±77	>20 000	>20 000	–	–
**14 b**		‐H	12 834±2469	1237±142	>20 000	0.1	–
**16 b**		‐acryl	1140±576	119±9	16 316±4855	0.1	14
**13 c**	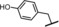	‐H	5194±1587	>20 000	>20 000	–	–
**15 c**		‐acryl	381±116	>20 000	>20 000	–	–
**14 c**		‐H	15 590±3125	>20 000	17 701±4069	–	1
**16 c**		‐acryl	813±159	5033±1061	1277±233	6	2

In addition to determination of the IC_50_ values, ligands that showed a promising activity toward Akt1 or Akt2 (IC_50_ <500 nm) were further characterized with respect to their binding kinetics, by determining the *K*_i_ and *k*_inact_. The evaluation of *k*_inact_/*K*_i_, a parameter that combines both the reversible affinity and the rate of covalent bond formation, helped to elucidate the exact contributions of the presented electrophilic warheads (Table 2, Table 3). The kinetic data indicated that in the case of Akt1, the rate of covalent bond formation contributes to the potency of the ligand. All three molecules with modifications at the 6′‐position seemed to bind in a more ideal orientation with respect to the electrophile being addressed by one of the cysteine thiols, whereas the 5′‐position modified ligands displayed a slower *k*_inact_ value. Compared to the well‐described borussertib, the pyrazinonic ligands lacked a potent reversible affinity, but indicated a similar rate of the covalent inactivation, resulting in the small values of *k*_inact_/*K*_i_. In the case of Akt2, the potency of borussertib and **16 b** relied more on the reversible binding step, while the covalent bond formation was slower compared to Akt1. However, the reactivity of the two cysteine residues that are known to be addressed in Akt1 have not been thoroughly characterized in Akt2 yet and need further investigation.14a

**Table 2:** Kinetic evaluation of a selected set of covalent‐allosteric Akt ligands with Akt1.

	Akt1^wt^		
#	*K*_i_ [nM]	*k*_inact_ [10^3^ min^−1^]	*k*_inact_/*K*_i_ [10^3^ μM^−1^ s^−1^]
borussertib	3±0.4	114±10	755±68
**15 a**	71±1	114±12	28±3
**16 a**	59±4	84±3	24±2
**15 b**	1243±209	138±13	2±0.2
**16 b**	1432±333	95±29	1±0.4
**16 c**	755±227	113±32	3±0.2

**Table 3:** Kinetic evaluation of covalent‐allosteric Akt ligands with Akt2.

	Akt2^wt^		
#	*K*_i_ [nM]	*k*_inact_ [10^3^ min^−1^]	*k*_inact_/*K*_i_ [10^3^ μM^−1^ s^−1^]
borussertib	30±5	34±6	19±1
**16 b**	57±6	30±5	9±1

Orthogonal evidence for the covalent bond formation was provided through analysis of the pyrazinone ligands together with Akt1 or Akt2 in mass spectrometry experiments (Figure 2). All spectra showed a mass shift in comparison to the apo protein with mass differences corresponding to the mono‐labeled protein with the specific ligand. The covalent modification of Akt2 is shown here for the first time and was further validated through MS/MS‐analysis, which revealed selective labeling of the targeted cysteine residues (Figure S3). Together with the activity‐based analysis of the ligands, these experiments provide additional proof for the selective covalent modification of the target proteins, either Akt1 or Akt2.

**Figure 2:**
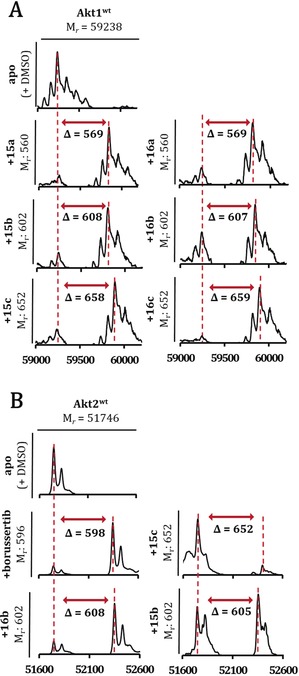
Deconvoluted mass spectra of A) Akt1^wt^ and B) Akt2^wt^ after incubation with DMSO (apo) and selected regioisomeric covalent‐allosteric Akt ligands. All tested molecules show mass differences according to a mono‐labeling of the protein and the completeness complies with the potency of the ligands for the specific isoform. Mass spectra were recorded using denaturing conditions.

To acquire more insights into the exact binding mode of the novel ligands, we co‐crystallized the two pyrazinones **16 a** and **15 c** with full‐length Akt1 (Figure 3). The complex structures demonstrate the anticipated binding mode and show that both ligands stabilize the full‐length protein in the inactive PH‐in conformation. Most interactions are comparable to the previously described set of co‐crystal complexes.14b The western molecule part is essentially stabilized by π–π‐stacking with Trp 80 and Tyr 272 (Figure S5). The complex with **15 c** displays that the presented hydroxyphenyl ring is in close contact to the PH‐domain interface and a shift in the loop around Ser 205 occurs, resulting in an interaction of the hydroxy sidechain with Lys 268 instead of Gln 203. The data suggests that both inhibitors can label Cys 310 and Cys 296, which might be based on a slightly different orientation of the electrophilic warhead (Figure S4). Additional studies with full‐length Akt2 and Akt3 are ongoing and will lead to a better structural understanding and defined insights into the exact constitution of the specific binding pockets.

**Figure 3:**
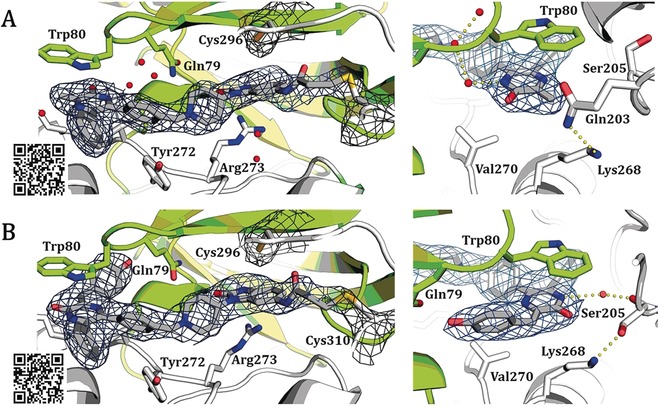
Co‐crystal structures of full‐length Akt1 in complex with novel covalent‐allosteric inhibitors, 2F_O_−F_C_ maps contoured at 0.8 σ. A) Co‐crystal structure of Akt1 with **16 a** (PDB: 6S9W) and B) co‐crystal structure of Akt1 with **15 c** (PDB: 6S9X). The electron density indicates possible covalent bond formation with both Cys 296 and Cys 310 but suggests preferred modification of Cys 310 (see mFo−DFc simulated annealing omit maps in Figure S4).

The molecules presented in this study are intended to serve as probes to dissect the function of the individual isoforms and thus must retain their selectivity profile within the cellular environment. To test this proposal, based on the biochemical and structural evaluation, the novel covalent‐allosteric ligands were also analyzed regarding their selectivity towards the Akt isoforms in a cellular context. For this purpose, the phosphorylation states of Akt2 were investigated in immunoblotting experiments (Figure 4) and, in addition, the set of ligands was tested in a cell viability assay (Table S2). These studies displayed a concentration‐dependent downregulation of the specific isoform phosphorylation level after 24 h treatment, showing that the molecules can retain their efficacy even in complex cellular systems. Further studies are underway to elucidate the consequences of a downregulated Akt2 phospho‐signal.

**Figure 4:**
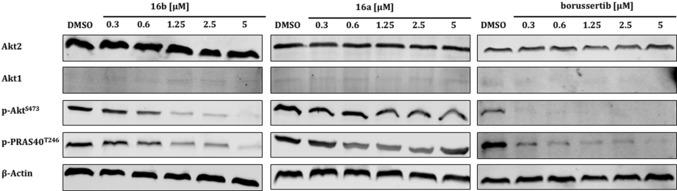
In vitro immunoblotting experiments. Western blot analyses for cancer cell line PANC1 with Akt1 knock‐out treated with indicated doses of CAAIs for 24 hours demonstrating dose‐dependent downregulation of *p*‐AKT^S473^ and phosphorylation of downstream target *p*‐PRAS40^T246^, consistent with the observed biochemical potency of the ligands for Akt2.

Approaching the protein kinase Akt with covalent‐allosteric ligands helped to identify specific binding capacities and preferences of the three isoforms. Structural homology model analyses in combination with sequence alignments revealed possible orientation‐dependent differences within the isoform binding pockets. These outcomes guided the design of a diverse set of ligands with modifications at either the 5′‐ or 6′‐position. The three introduced moieties, as well as the derivatized position, showed great variations in the activity‐based evaluations. In particular, the 5′‐modified ligands were less favored by Akt1 than the 6′‐position derivatives. For Akt2 and Akt3, the data displayed preferential binding of the 5′‐substituted molecules, nicely correlating with the predictions from the homology model that indicated a less rigid helical structure on this site of the isoform pockets. In general, activities of these pyrazinones towards Akt3 were moderate. Previously identified allosteric ligands exhibited a limited potency as well, indicating a less favorable formation of the interdomain binding pocket or a shifted conformational equilibrium towards the PH‐out conformation, similar to the Akt^E17K^ mutant.^20^ Furthermore, we demonstrated the irreversible modification through MS experiments and characterized the covalent bond formation of the CAAIs with kinetic studies in Akt1 and, for the first time, in Akt2. Cellular characterization helped to investigate the possible translation of identified selectivity profiles into a more complex system. In particular, **16 a** and **16 b** function as promising candidates for further functionalization, retaining their selective modification towards Akt1 and Akt2. However, to serve as molecular probes in perturbation studies, additional parameters concerning functionalization and bioavailability need to be evaluated more thoroughly.^21^

In summary, we have exploited the concept of covalent‐allosteric inhibitors as tools for an isoform‐selective modification of the protein kinase Akt. A structural and sequence comparison model was developed for the design of small molecules with the right characteristics to perturb the isoform binding pockets in a diverse and orientation‐guided manner. Biochemical characterization, kinetic evaluation, as well as protein crystallography led to a more thorough understanding of the Akt isoforms and the structure–activity relationship of the covalent‐allosteric ligands in the context of isoform selectivity. These insights set a starting point for a more elaborate structure‐guided development of chemical probes, which would help to analyze the isoform functions in context of health and disease states in living organisms and result in a step forward towards functional molecules that can lead to new therapeutic advantages.21b

## Conflict of interest

The authors declare no conflict of interest.

## Supporting information

As a service to our authors and readers, this journal provides supporting information supplied by the authors. Such materials are peer reviewed and may be re‐organized for online delivery, but are not copy‐edited or typeset. Technical support issues arising from supporting information (other than missing files) should be addressed to the authors.

Supplementary
