# Supplementary material for: Covalent‐Allosteric Inhibitors to Achieve Akt Isoform‐Selectivity
Source: Angew Chem Int Ed Engl. 2019 Nov 8;58(52):18823–9. doi: 10.1002/anie.201909857 (PMC6972997; doi:10.1002/anie.201909857)
Supplement: Supplementary file 1 — Supplementary [file ANIE-58-18823-s001.pdf]

Angewandte Chemie
Eine Zeitschrift der Gesellschaft Deutscher Chemiker

Supporting Information

Covalent-Allosteric Inhibitors to Achieve Akt Isoform-Selectivity

*Lena Quambusch, Ina Landel, Laura Depta, Jörn Weisner, Niklas Uhlenbrock, Matthias P. Müller, Franziska Glanemann, Kristina Althoff, Jens T. Siveke, and Daniel Rauh**

anie_201909857_sm_miscellaneous_information.pdf

Table of Contents

Experimental procedures **3**
Chemistry 3
Sequence Alignment and Homology Model 3
Activity-based Assay for IC_{50}-Determination and Kinetic Characterization 3
Mass spectrometry 3
Protein Expression, Purification, and Crystallization 3
Cell Culture and Viability Assay 4
CRISPR/Cas9-mediated knockout of AKT1/2 4
Western Blots Analysis 4
Antibodies 5
Data Availability 5
Synthetic procedures and compound characterization 5
Results and Discussion **10**
Figure S1. Sequence Alignment 10
Figure S2. Homology Model 10
Figure S3. MS/MS Analysis 11
Figure S4. Simulated annealing refinement 12
Figure S5. Protein-ligand interaction plots with OEChem/OEDepict 13
Table S1. Data collection of X-ray refinement 14
Table S2. Cellular Evaluation 15
Figure S6. Western blot 15
NMR Spectra (Figure S7-S28) 16
References **38**
Author Contributions **38**

Experimental Procedures

Chemistry

All reagents and solvents were purchased from Acros, Activate Scientific, Alfa Aesar, Apollo Scientific, Merck, Sigma-Aldrich, TCI Chemicals or VWR and used without further purification. Dry solvents were purchased as anhydrous reagents from commercial suppliers. ^{1}H and ^{13}C NMR spectra were recorded on a Bruker Avance DRX AV400 (400 MHz and 101 MHz), AV500 (500 MHz and 125 MHz), AV600 (600 MHz and 151 MHz) and AV700 (700 MHz and 176 MHz). ^{1}H chemical shifts are reported in δ (ppm) as s (singlet), d (doublet), dd (doublet of doublet), t (triplet), q (quartet), m (multiplet) and b (broad singlet) and are referenced to the residual solvent signal: $CDCl_3$ (7.26), DMSO-d_6 (2.50) or MeOD-d_4 (3.34). ^{13}C spectra are referenced to residual solvent signal: $CDCl_3$ (77.1), DMSO-d_6 (39.52) or MeOD-d_4 (49.86). High-resolution electrospray ionization mass spectra (ESI-FTMS) were recorded on a Thermo LTQ Orbitrap (high-resolution mass spectrometer from Thermo Electron) coupled to an Accela HPLC system supplied with a Hypersil GOLD column (Thermo Electron). LC-MS (ESI-MS) analysis was performed using Agilent HPLC system (1100 series) with CC 125/4 Nucleodur C18 gravity column (3 µm) from Macherey Nagel coupled to a Thermo Scientific Finnigan LCQ Advantage Max Ion Trap and ESA Corona detector. Analytical TLC was carried out on Merck 60 F254 aluminium-backed silica gel plates. Compounds were purified by column chromatography using VWR silica gel (40 - 63 µm particle size) or flash chromatography on a Biotage Isolera One using Büchi Reveleris Silica Cartridges (4 - 120 g) monitored by UV at λ = 210 nm and 280 nm. Preparative HPLC was conducted on an Agilent HPLC system (1200 series) with a VP 125/21 Nucleodur C18 column from Macherey-Nagel and monitored by UV at λ = 210 nm and 254 nm. All final compounds were purified to > 95 % purity as determined by high-performance liquid chromatography (HPLC). Purity was measured using Agilent 1200 series HPLC systems with UV detection at λ = 210 nm (system: Agilent Eclipse XDB-C18 4.6 mm x 150 mm, 5 µM, 10 – 100 % MeCN in H_2O, with 0.2 % TFA, for 15 min at 1.0 mL/min).

Sequence Alignment and Homology Model

The sequence alignment of the three Akt isoforms was performed with Clustal Omega[1] based on the following sequence Input files: Akt1_P31749, Akt2_P31751, Akt3_Q9Y243. Homology models for Akt2 and Akt3 were generated with the SWISS-MODEL homology-modelling server based on a Akt1 full-length co-crystal structure (PDB: 6HHI) and the specific sequence files (as mentioned above).[2]

Activity-based Assay for IC_{50}-Determination and Kinetic Characterization

The biochemical half maximal inhibitory concentrations (IC_{50}) were determined with the HTRF KinEASE assay (Cisbio) as previously described.[3] The obtained data were normalized to positive (1 µM staurosporine) and negative (DMSO) control and subsequently fit to a four-parameter logistic model using the Quattro Software Suite (quattro research). Time-dependent IC_{50}-measurements were performed as described. Briefly, IC_{50} values were determined for twelve different incubation times and afterwards analyzed according to literature procedure using XLfit (Version 5.4.0.8, IDBS, Germany).[4]

Mass spectrometry

We used Akt1 and Akt2 for MS experiments and incubated 10 µM of the protein with 100 µM of the inhibitor in a buffer for 1 h and in case of Akt2 4 h. We analyzed the samples by mass spectrometry using a Thermo Fisher Scientific Ultimate 3000 HPLC system connected with a Thermo Fisher Scientific Velos Pro (2D ion trap). The sample (5 µL) was injected and separated by an AdvanceBio Desalting-RP Cartridge (Agilent Technologies) starting at 95 % solvent A (0.1 % formic acid in water) and 5 % solvent B (0.1 % formic acid in acetonitrile) for 30 seconds, followed by a linear gradient over 2.5 minutes up to 80 % solvent B. A mass range of 700-2000 m/z was scanned and raw data were deconvoluted and analyzed with ProMass for Xcalibur (Novatia). The deconvoluted mass spectra were smoothed and cropped to a mass range of 59,000 – 60,300 m/z with the software mMass (version 5.5.0).[5]

ESI-MS/MS samples were prepared accordingly prior to SDS-PAGE, treatment of the gel pieces (washing, reduction, alkylation) and tryptic digest.[6] Subsequently, samples were thawed, dissolved in 20 µL of 0.1 % TFA in water, sonicated at room temperature for 15min, and centrifuged at 15000 x g for 1min shortly before analysis. 3 µL of sample were loaded onto a pre-column cartridge and desalted for 5 min using 0.1 % TFA in water as eluent at a flow rate of 30 µL/min. The samples were back-flushed from the pre-column to the nano-HPLC column during the whole analysis. Elution was performed using a gradient starting at 5 % B with a final composition of 30 % B after 35min (flow rate 300 nL/min) using 0.1% formic acid in water as eluent A and 0.1% formic acid in acetonitrile as eluent B and a column temperature of 40 °C. The nano-HPLC column was washed by increasing the percentage of solvent B to 60 % in 5 min and to 95 % in additional 5 min, washing the columns for further 5 min, flushing back to starting conditions and equilibration of the system for 14 min. During the complete gradient cycle, a typical TOP10 shot-gun proteomics method for the MS and MS/MS analysis was used. For full scan MS experiments a mass range of m/z 300 to 1650 was scanned with a resolution of 70000. MS/MS experiments were followed by up to ten high energy collision dissociation (HCD) MS/MS scans with a resolution of 17500 of the most intense at least doubly charged ions. Data evaluation was performed using MaxQuant.[7] Spectra were searched against the specific Akt sequence and a contamination database using a false discovery rate of 1% on peptide and protein level using a decoy database for determination of the false discovery rate. For database search oxidation of methionine and N-terminal acetylation of proteins, carbamidomethylation of cysteines, and artificial modification of cysteines were defined as variable modifications.

Protein Expression, Purification, and Crystallization

The following genes encoding either for Akt1(2-446, E114/115/116A) or Akt2(2-447) including an N-terminal His6-Tag followed by a TEV protease recognition site were synthesized by GeneArt AG (Regensburg, Germany) and cloned into the pIEx/Bac3 expression

vector (Merck Millipore) using NcoI and BamHI restriction sites. Transfection, virus generation, and amplification as well as protein expression were carried out in Spodoptera frugiperda (Sf9) cells (ThermoScientific) following the BacMagic protocol (Merck Millipore). Infected insect cells were grown in Erlenmeyer flasks for 72 hours at 27 °C with shaking at 120 rpm, subsequently harvested by centrifugation at 3,000 x g for 15 min and washed once with PBS before being flash frozen in liquid nitrogen. Afterwards, cells were thawed and resuspended in lysis buffer (50 mM Tris, 500 mM NaCl, 1 mM DTT, 10 % glycerol, 0.1 % Triton X-100, pH 8.0, EDTA-free protease inhibitor cocktail (Sigma)). Cells were lysed using a microfluidizer, the lysate was cleared by centrifugation (40,000 x g, 1 h). The supernatant was loaded onto a Ni-NTA Superflow cartridge (Qiagen). Bound protein was eluted in buffer containing 50 mM Tris, 500 mM NaCl, 500 mM imidazole, 1 mM DTT, 10 % glycerol, pH 8.0. For cleavage of the hexahistidine-tag, TEV protease was added to the pooled elution fractions and dialyzed overnight into buffer containing 25 mM Tris, 50 mM NaCl, 1 mM DTT, 5 % glycerol, pH 8.0 at 4 °C. The cleaved protein was further purified by anion-exchange chromatography using a HiTrap Q HP column (GE Healthcare) followed by size-exclusion chromatography on a HiLoad 16/60 Superdex 75 pg column (GE Healthcare) using buffer containing 50 mM HEPES, 200 mM NaCl, 1 mM DTT, 10% glycerol, pH 7.3. Afterwards, the protein was transferred into the storage buffer (25 mM Tris, 100 mM NaCl, 1 mM DTT, 10 % glycerol, pH 7.5) using a Superdex 75 10/300 GL column (GE Healthcare), concentrated and stored at -80 °C.
For crystallization, purified protein at a concentration of 3 mg/mL was incubated with 3 eq. of every inhibitor on ice for 60 min. The samples were centrifuged at 20,000 x g for 10 min before hanging drops were prepared in 15-well crystallization plates (EasyXtal Tool, Qiagen) by mixing protein-ligand complex with reservoir solution (1:1) containing 1.25 mM sodium acetate pH 5.2, 3.75 mM sodium citrate 7.0, 24 % PEG MME 2000 for Akt1 in complex with **16a** and pH 7.5, 15% PEG MME 2000 for Akt1 in complex with **15c** at 20 °C. Diffraction-grade crystals grew within 3 days and were cryoprotected using 20 % ethylene glycol before they were flash cooled in liquid nitrogen. X-ray diffraction data were collected at the PXII X10SA beam line of the Swiss Light Source (PSI, Villigen, Switzerland) with wavelengths close to 1 Å. The diffraction data were integrated with XDS and scaled using the program XSCALE.[8] Due to ice rings for the crystal structure of Akt1 in complex with **16a**, the resolution ranges 3.93 Å - 3.87 Å, 3.70 Å - 3.64 Å, 3.47 Å - 3.41 Å, 2.70 Å - 2.64 Å and 2.28 Å - 2.22 Å were excluded during the data processing with XDS[8] leading to reduced overall data completeness of 91.8%. The crystal structure was solved by molecular replacement with PHASER using an unpublished co-crystal structure of Akt1 in complex with another covalent-allosteric inhibitor as template.[9] The manual modification of the molecule of the asymmetric unit was performed using the program COOT[10] and with the help of the Dundee PRODRG server the inhibitor topology files were generated.[11] As shown before for covalent-allosteric Akt inhibitors by ESI-MS/MS analysis, due to modification of both Cys296 and Cys310, the linker region of the covalent bond is not fully resolved in the structures.[12] However, the covalent bond formation was modeled towards Cys310 for both inhibitors as the mFo-DFc maps suggest preferred labeling of this cysteine over Cys296. For multiple cycles of refinement PHENIX.refine[13] was employed and the final structure was evaluated by Ramachandran plot analysis using MolProbity.[14] Validation and optimization of the model was performed with help of the PDB_REDOserver[15] and crystal structures were visualized using PyMOL (See also Supplement Fig. S3).[16]

Cell Culture and Viability Assay

Source and cultivation of all tested cell lines was described in detail elsewere.[17] PANC1 cell line were obtained from the American Type Culture Collection (ATCC). Cell lines were cultured in DMEM medium (Gibco) supplemented with 10% fetal bovine serum (FBS) (PAN-Biotech) and 1% penicillin/streptomycin (Gibco). Cells were cultured in a humidified incubator at 37 °C, 5% CO_2.
For cell viability analysis, cells were plated on day 0 into white 384-well cell culture plates (Greiner Bio-One) using a Multidrop reagent dispenser (Thermo) at cell numbers that ensure linear and optimal luminescent signal intensity (AN3-CA: 800 cells/well; T-47D: 800 cells/well; ZR-75-1: 400 cells/well). Then the cells were incubated for 24 h in a humidified atmosphere at 37 °C/5 % CO_2. The cells were treated with inhibitors in serial dilutions ranging from 30 µM down to 0.1 nM using an Echo 520 acoustic liquid handler (Labcyte Inc.). Cell viability was analyzed on day 5 using the CellTiter-Glo assay (Promega) as per manufacturer's instructions. Luminescence signal was recorded by an EnVision Multilabel 2104 Plate Reader (PerkinElmer) with 500 ms integration time. All data were normalized to the plate positive control (30 µM staurosporine) and negative control (DMSO). Finally, the data was processed with the Quattro Software Suite using a four-parameter logistic model. As a quality control, the Z'-factor was calculated from 16 positive and negative control values. Only assay results showing a Z'-factor ≥ 0.5 were used for further analysis. All experimental points were measured in duplicates for each plate and were replicated in at least three times.

CRISPR/Cas9-mediated knockout of AKT1/2

PANC1 cells were first stably transduced with lentiviral particles produced in HEK293T cells using the following vectors: psPAX2 (Addgene, #12260) and pCMV-VSV-G (Addgene, #8454), lentiCas9-Blast (Addgene, #52962) and Lipofectamine LTX and Plus Reagent (Life Technologies). Cells were selected by growth in the presence of 7,5 µg/ml blasticidin and surviving colonies were analyzed for successful CAS9 expression by Western blot. PANC1-Cas9 cells were then seeded in 6-well plates (2.5 x 10^5) to reach a confluency of 70-80% at the day of transduction. For performing AKT1 and AKT2 knockdown, 1.5 µg of respectively two sgRNAs targeting AKT1 (Addgene, #75500) and AKT2 (Addgene, #77505) were used for producing lentiviral particle (description before). Seeded PANC1-Cas9 cells were transduced with those lentiviral particles and selected by growth in presence of 1 µg/ml of puromycin for 14 days. Surviving colonies were analyzed for successful knockdown by Western blot and qRT-PCR (see Fig. S5).

Western blot analysis

Cells were seeded into six-well tissue culture plates (Sarstedt) yielding 70-90% confluency after overnight incubation. Afterwards, cells were treated with various concentrations of inhibitors or DMSO and incubated for additional 24 h before cells were washed twice with

ice-cold PBS. Cell lysis was initiated by addition of 100 µL RIPA buffer (Cell Signaling Technology) per well supplemented with phosphatase and protease inhibitor cocktails (Sigma) followed by incubation on ice for 30 min. Cells were then harvested by scraping and transferred into pre-cooled microcentrifuge tubes. Whole cell lysates were cleared by centrifugation at 14,000 x *g*/4 °C for 10 min and transferred into fresh, precooled microcentrifuge tubes. Protein concentrations were determined using the Pierce BCA protein assay (Thermo) as per manufacturer's instructions. Equal amounts of protein were separated by SDS-PAGE and transferred to Immobilon-FL PVDF membranes (Merck Millipore) using Pierce™ 1-step transfer buffer (Thermo) and the Pierce™ Power Blotter (Thermo). Membranes were washed for 5 min with ddH_2O, blocked with Odyssey® Blocking Buffer TBS (Li-Cor) for 1 h at room temperature and then incubated with primary antibodies diluted in Odyssey® Blocking Buffer TBS overnight at 4 °C with gentle agitation. On the next day, the membranes were washed three times with TBS-T (50 mM Tris, 150 mM NaCl, 0.05% Tween 20, pH 7.4) for 5 min before being incubated with secondary antibodies diluted in Odyssey® Blocking Buffer TBS for 1 h at room temperature with gentle agitation. Finally, the membranes were washed three times for 5 min with TBS-T and then scanned using an Odyssey® CLx imaging system (Li-Cor).

Antibodies

Anti-pAkt (Ser473) (CST, cat. no. 4060), anti-tAkt1 (CST, cat. no. 2938), anti-tAkt2 (CST, cat. no. 3063), anti-pPRAS40(Thr246) (CST, cat. no. 2997), anti-β-Actin (Sigma, cat. no. A5441), anti-mouse IgG (H+L) (DyLight™ 680 Conjugate) (CST, cat. no. 5470), anti-rabbit IgG (H+L) (DyLight™ 800 4X PEG Conjugate) (CST, cat. no. 5151).

Data Availability

The structures of Akt1 in complex with the inhibitors **15c** and **16a** have been deposited in the Protein Data Bank under PDB-ID 6S9X for **15c** and PDB-ID 6S9W for **16a**. 3D structural models for Augment are also available via QR-codes within the corresponding figures.[18]

Synthetic procedures and compound characterization

Common Procedure A: Pyrazinone cyclization

In a round bottom flask 1.0 eq. of the diketone and 3.0 eq. of an α-amino carboxamide were dissolved in a solvent mixture of ethanol and acetic acid (20:1, 3 mL/mmol). The suspension was heated under reflux conditions for 24 h at 90 °C. The reaction mixture was treated with few mL of 5 M NaOH solution and stirred for additional 12 hours at rt. The solution was extracted with dichloromethane, the combined organic fractions dried over Na_2SO_4 and the solvent was evaporated *in vacuo*. The pure product was obtained after silica gel column chromatography (7 - 10 % MeOH/DCM + 1 % NH_3).

Common Procedure B: Boc deprotection

A solution of 1.0 eq. boc-protected amine was dissolved in 1,4-dioxane (5mL/mmol) and after addition of 4 N HCl in 1,4-dioxane (2.5 mL/mmol) the mixture was stirred for 12 h at room temperature. After completion, the reaction mixture was basified with 10 M NaOH solution. The resulting precipitate was separated and extracted with dichloromethane. The combined organic fractions were dried over Na_2SO_4 and the solvent was evaporated *in vacuo*. The pure product was obtained after silica gel column chromatography (7 - 10 % MeOH/DCM + 1 % NH_3).

Common Procedure C: Acrylamide coupling

First 1.0 eq. of the aniline was dissolved in dry THF (10 mL/mmol), 3 eq. DIPEA were added and the solution was stirred for 15 min at 0 °C under argon atmosphere. Subsequently, a solution of 1.5 eq. acryloyl chloride in few mL THF was added dropwise. The reaction mixture was stirred at room temperature until completion. Afterwards the reaction was quenched with a saturated solution of $NaHCO_3$. The aqueous layer was extracted with DCM and the combined organic layers were washed with saturated NaCl solution and dried over Na_2SO_4. The solvent was evaporated *in vacuo* and silica gel column chromatography yielded the desired product (7 - 10 % MeOH/DCM + 1 % NH_3).

Synthesis of 5-chloro-6-nitro-1-(piperidin-4-yl)-1,3-dihydro-2H-benzo[d]imidazol-2-one (**2**). 5-Chloro-1-(piperidin-4-yl)-1,3-dihydro-2*H*benzo[*d*]imidazol-2-one (**1**, 2.5 g, 9.9 mmol) was stirred at 60 °C in 100 mL *o*-xylene. Then, 6 eq. of concentrated nitric acid (69 %) were added dropwise and the reaction mixture was heated for 1 h. The solvent was decanted and the residual crude product was washed with MeOH. Afterwards, the pure product was filtered off and yielded the title compound as pale yellow solid **2** (2.80 g, 9.4 mmol, 95 %).**^{1}H-NMR** (500 MHz, DMSO-*d$_6$*) δ ppm 1.92 (d, *J* = 12.51 Hz, 2H) 3.07 (q, *J* = 11.75 Hz, 2H) 3.46 (d, *J* = 12.21 Hz, 2H) 4.53 - 4.65 (m, 1H) 7.28 (s, 1H) 8.07 (s, 1H) 8.36 (s, 1H) 8.58 (s, 1H) 11.78 (s, 1H); **^{13}C-NMR** (126 MHz, DMSO-*d$_6$*) δ ppm 25.20 (s, 2C) 43.06 (s, 2C) 47.66 (s, 1C) 106.13 (s, 1C) 110.64 (s, 1C) 119.38 (s, 1C) 128.14 (s, 1C) 133.10 (s, 1C) 140.65 (s, 1C) 153.84 (s, 1C); **HPLC-MS (ESI):** [R_t]: 2.45 min, *m/z* for $C_{12}H_{13}N_4O_3Cl$ ([M+H$^+$]), 296.71 calcd., 297.31 found.

Synthesis of 6-amino-1-(piperidin-4-yl)-1,3-dihydro-2H-benzo[d]imidazol-2-one (**3**). 5-Chloro-1-(piperidin-4-yl)-1,3-dihydro-2*H*-benzo[*d*]imidazol-2-one (**2**, 4.7 g, 15.9 mmol, 1.0 eq.), 5 % Pd/C moistened with water (0.2 g) and ammonium formate (10.0 g, 159 mmol, 10 eq.) were dissolved in methanol (48 mL, 3 mL/mmol) and allowed to stir for 12 h at 80 °C. The reaction mixture was filtered over Celite, evaporated and the crude product was used without further purification. **^{1}H-NMR** (500 MHz, MeOD-*d$_4$*) δ ppm 2.06 (d, *J* = 13.73 Hz, 2H) 2.82 (qd, *J* = 13.22, 4.12 Hz, 2H) 3.25 - 3.29 (m, 1H) 3.32 - 3.34 (m, 1H) 3.60 (d, *J* = 12.82 Hz, 2H) 4.69 (tt, *J* = 12.25, 4.01 Hz, 1H) 7.12 (dd, *J* = 8.32, 1.91 Hz, 1H) 7.17 - 7.21 (m, 1H) 7.54 (d, *J* = 1.68 Hz, 1H) 8.81 (s, 1H) 11.09 (s, 1H). **^{13}C-NMR**

(126 MHz, MeOD-d_4) δ ppm 26.97 (s, 2C) 45.06 (s, 2C) 49.82 (s, 1C) 105.34 (s, 1C) 111.40 (s, 1C) 117.38 (s, 1C) 125.65 (s, 1C) 130.22 (s, 1C) 131.46 (s, 1C) 156.34 (s, 1C); **HPLC-MS (ESI):** [R_t]: 0.74 min, *m/z* for $C_{17}H_{24}N_4O_3$ ([M+H$^+$]), 233.29 cacld., 233.28 found.

Synthesis of tert-butyl(2-oxo-3-(piperidin-4-yl)-2,3-dihydro-1H-benzo[d]imidazol-5-yl)-carbamate (**4**)**.** 6-Amino-1-(piperidin-4-yl)-1,3-dihydro-2*H*-benzo[*d*]imidazol-2-one (**3**, 3.7 g, 15.9 mmol, 1.0 eq.) was stirred in 40 mL 1,4-dioxane (2.5 mL/mmol) and 10 % acetic acid. 1.5 eq. Boc_2O (4.7 mL, 23.9 mmol) was dissolved in 10 mL 1,4-dioxane and then added dropwise to the reaction mixture, which was allowed to stir overnight at room temperature. The mixture was neutralised with 10 M NaOH and extracted with DCM. The combined organic fractions were dried over Na_2SO_4 and evaporated *in vacuo*. Silica gel column chromatography (7 - 10 % MeOH/DCM + 1 % NH_3) yielded the title compound as a light yellow solid **4** (1.33 g, 4.02 mmol, 25 % over 2 steps). **^{1}H-NMR** (600 MHz, DMSO-d_6) δ ppm 1.47 (s, 9 H) 1.58 (d, *J* = 11.55 Hz, 2H) 2.07 - 2.21 (m, 2 H) 2.57 (t, *J* = 11.46 Hz, 2H) 3.07 (d, *J* = 11.74 Hz, 2H) 4.13 (tt, *J* = 12.50, 7.30 Hz, 1H) 6.83 (d, *J* = 8.25 Hz, 1H) 6.96 (s, 1H) 7.51 (s, 1H) 8.52 - 8.56 (m, 1H) 9.08 - 9.19 (m, 1H) 10.60 - 10.73 (m, 1H); **^{13}C-NMR** (151 MHz, DMSO-d_6) δ ppm 28.17 (s, 3C) 30.02 (s, 2C) 45.85 (s, 2C) 48.59 (s, 1C) 50.50 (s, 1C) 78.58 (s, 1C) 108.49 (s, 2C) 123.46 (s, 1C) 129.11 (s, 1C) 132.99 (s, 1C) 152.98 (s, 1C) 153.97 (s, 1C); **HPLC-MS (ESI):** [R_t]: 2.69 min, *m/z* for $C_{17}H_{24}N_4O_3$ ([M+H$^+$]), 333.40 calcd., 333.28 found.

Synthesis of 1-methyl-4-(phenylethynyl)benzene (**7**)*.* Under argon atmosphere diisopropylamine (5 mL, 1mL/mmol) was degassed for 10 min and then iodobenze (546.4 µL, 4.90 mmol, 1.0 eq.), tetrakis(trisphenylphosphine)palladium(0) (7.0 mg, 0.05 mmol, 0.1 eq.) and copper(I)iodide (9.3 mg, 0.05 mmol, 1.0 eq.) were added subsequently. Then, tolylacytelene (704,1 µL, 5.88 mmol, 1.2 eq.) was added dropwise and the reaction mixture stirred for 12 h at 60 °C. The resulting suspension was extracted with EtOAc and the combined organic fraction washed with saturated NH_4Cl solution, dried over Na_2SO_4 and evaporated *in vacuo*. Silica gel column chromatography (PE/EtOAc, 100/1) yielded the titled compound as a white solid **7** (940 mg, 4.89 mmol, 98 %). R_f (**7**) = 0.52 (PE/DCM 4/1); **^{1}H-NMR** (500 MHz, DMSO-d_6) δ ppm 2.34 (s, 3H) 7.24 (d, *J* = 7.48 Hz, 2H) 7.42 (d, *J* = 4.88 Hz, 3 H) 7.45 (d, *J* = 7.78 Hz, 2H) 7.54 (d, *J* = 4.27 Hz,2H); **^{13}C-NMR** (126 MHz, DMSO-d_6) δ ppm 21.07 (s, 1C) 88.73 (s, 1C) 89.51 (s, 1C) 119.24 (s, 1C) 122.45 (s, 1C) 127.10 (s, 1C) 129.01 (s, 2C) 129.42 (s, 2C) 131.31 (s, 4C) 138.61 (s, 1C).

Synthesis of 1-phenyl-2-(p-tolyl)ethane-1,2-dione (**8**)*.* First, an aqueous oxidation buffer (100 mL) was assembled with $MgSO_4$ (4.06 g, 33.8 mmol, 8.8 eq.) and $NaHCO_3$ (838 mg, 10.0 mmol, 2.6 eq.) The acetylene **7** (737 mg, 3.8 mmol, 1.0 eq.) was dissolved in a mixture of acetone (60 mL, 15 mL/mmol) and oxidation buffer (20 mL, 5 mL/mmol), then potassium permanganate (2.42 g, 15.4 mmol, 4.0 eq.) was added in one portion. The mixture was allowed to stir for 4 h at room temperature. After completion the reaction was quenched with addition of $NaNO_2$ (1.0 g) in small portions as well as 10 % aqueous solution of sulphuric acid (20 mL). Followed by extraction with EtOAc the combined organic fractions were washed with Brine, then dried over Na_2SO_4 and evaporated *in vacou*. Silica gel column chromatography (Pe/EtOAc, 50/1) yielded the titled compound **8** (738 mg, 3.29 mmol, 86 %) as a yellow oil. **^{1}H-NMR** (500 MHz, DMSO-d_6) δ ppm 2.42 (s, 3H) 7.44 (d, *J* = 8.09 Hz, 2H) 7.44 - 7.44 (m, 1H) 7.61 - 7.65 (m, 2H) 7.77 - 7.83 (m, 3H) 7.90 (d, *J* = 7.78 Hz, 2H); **^{13}C-NMR** (126 MHz, DMSO-d_6) δ ppm 21.47 (s, 1C) 129.55 (s, 4C) 129.73 (s, 2C) 129.84 (s, 1C) 130.10 (s, 2C) 132.31 (s, 1C) 135.52 (s, 2C) 194.50 (s, 1C) 195.03 (s, 1C); **HPLC-MS (ESI):** [R_t]: 6.68 min, *m/z* for $C_{17}H_{24}N_4O_3$ ([M]), 224.26 calcd., 224.92 found.

Synthesis of 1-(4-(bromomethyl)phenyl)-2-phenylethane-1,2-dione (**9**)*.* The diketone **8** (0.39 g, 1.72 mmol, 1.0 eq.) and N-bromsuccinimide (0.34 g, 1.89 mmol, 1.1 eq.) were dissolved in tetrachloromethane (10 mL, 5.5 mL/mmol) and at last the initiator benzoylperoxide (60.0 mg, 0.34 mmol, 0.2 eq.) was added. The mixture was heated under reflux for 30 min. Afterwards the succinimide was filtered off and the solvent evaporated. Silica gel column chromatography (PE/EtOAc, 50 /1) yielded the desired compound as yellow solid **9** (0.47 g, 1.55 mmol, 90 %). R_f (**9**) = 0.46 (PE/EtOAc, 9/1); **^{1}H-NMR** (600 MHz, DMSO-d_6) δ ppm 4.80 (s, 2H) 7.62 - 7.66 (m, 2H) 7.70 (d, *J* = 8.07 Hz, 2H) 7.81 (t, *J* = 7.52 Hz, 1H) 7.94 (dd, *J* = 7.70, 3.67 Hz, 4H); **^{13}C-NMR** (151 MHz, DMSO-d_6) δ ppm 32.77 (s, 1C) 129.48 (s, 2C) 129.66 (s, 2C) 130.07 (s, 2C) 130.25 (s, 2C) 131.80 (s, 1C) 132.16 (s, 1C) 135.56 (s, 1C) 145.66 (s, 1C) 194.13 (s, 1C) 194.56 (s, 1C).

Synthesis of tert-butyl (2-oxo-3-(1-(4-(2-oxo-2-phenylacetyl)benzyl)piperidin-4-yl)-2,3-dihydro-1H-benzo[d]imidazol-5-yl)carbamate (**10**)*.* Under argon atmosphere the boc-protected benzimidazolone **4** (0.39 mg, 1.18 mmol, 1.0 eq.) and the dione **9** (0.41 g, 1.42 mmol, 1.2 eq.) were dissolved in dry THF (15 mL, 12 mL/mmol). Then the base DIPEA (0.62 mL, 3.56 mmol, 3.0 eq.) was added and the mixture stirred for 3 h at rt. The resulting suspension was extracted with DCM and the combined organic fraction were washed with $NaHCO_3$ solution. After drying with Na_2SO_4, filtration and evaporation of the solvents, a silica gel column chromatography was performed (5-7 % MeOH/DCM + 1 % NH_3) which yielded the titled compound as a yellow solid **10** (545 mg, 0.97 mmol, 82 %). **^{1}H-NMR** (500 MHz, DMSO-d_6) δ ppm 1.45 (s, 9 H) 1.63 (d, *J* = 9.31 Hz, 2H) 2.12 - 2.21 (m, 2H) 2.27 - 2.38 (m, 2H) 2.94 (d, *J* = 10.83 Hz, 2H) 3.63 - 3.69 (m, 2H) 4.05 - 4.18 (m, 1H) 6.83 (d, *J* = 8.39 Hz, 1H) 7.00 (s, 1H) 7.52 (s, 1H) 7.59 - 7.67 (m, 4H) 7.77 - 7.83 (m, 1H) 7.88 - 7.97 (m, 4H) 9.14 - 9.26 (m, 1H) 10.70 (s, 1H); **^{13}C-NMR** (126 MHz, DMSO-d_6) δ ppm 28.20 (s, 3C) 28.52 (s, 2C) 49.73 (s, 1C) 52.75 (s, 2C) 61.11 (s, 1C) 78.66 (s, 1C) 108.65 (s, 2C) 123.40 (s, 1C) 128.98 (s, 1C) 129.56 (s, 4C) 129.65 (s, 2C) 129.72 (s, 2C) 131.10 (s, 1C) 132.29 (s, 1C) 133.11 (s, 1C) 135.59 (s, 2C) 147.39 (s, 1C) 153.00 (s, 1C) 154.01 (s, 1C) 194.52 (s, 1C) 194.98 (s, 1C); **HPLC-MS (ESI):** [R_t]: 4.03 min, *m/z* for $C_{32}H_{34}N_4O_5$ ([M+H$^+$]), 555.65 calcd., 555.44 found.

Synthesis of tert-butyl (3-(1-(4-(6-isobutyl-5-oxo-3-phenyl-4,5-dihydropyrazin-2-yl)benzyl)piperidin-4-yl)-2-oxo-2,3-dihydro-1H-benzo[d]imidazol-5-yl)carbamate (**11b**) *and tert-butyl (3-(1-(4-(5-isobutyl-6-oxo-3-phenyl-1,6-dihydropyrazin-2-yl)benzyl)piperidin-4-yl)-2-oxo-2,3-dihydro-1H-benzo[d]imidazol-5-yl)carbamate* (**12b**)*. Tert*-butyl (2-oxo-3-(1-(4-(2-oxo-2-phenylacetyl)benzyl)piperidin-4-yl)-

2,3-dihydro-1H-benzo[*d*]imidazol-5-yl)carbamate (**10**, 100 mg, 0.18 mmol, 1.0 eq.) and leucine amide (70,4 mg, 0.54 mmol, 3.0 eq.) were used following common procedure A and yielded the titled regioisomeric compounds as a pale yellow solids (**11b**/**12b**). **Yield (11b):** 25 %; **^{1}H-NMR** (500 MHz, Chloroform-*d*) δ ppm 1.06 (d, *J* = 6.56 Hz, 6H) 1.50 (s, 9 H) 1.53 (s, 1H) 1.77 (s, 2H) 2.38 (dt, *J* = 13.54, 6.73 Hz, 2H) 2.56 - 2.73 (m, 2H) 2.83 (d, *J* = 7.02 Hz, 2H) 2.97 - 3.29 (m, 2H) 3.73 (d, *J* = 7.02 Hz, 1H) 4.16 - 4.31 (m, 1H) 6.66 - 6.80 (m, 1H) 6.91 - 7.04 (m, 2H) 7.23 (d, *J* = 3.51 Hz, 3H) 7.28 - 7.41 (m, 5H) 7.44 - 7.58 (m, 4H) 7.97 (d, *J* = 1.68 Hz, 1H) 10.41 - 10.80 (m, 1H); **^{13}C-NMR** (126 MHz, Chloroform-*d*) δ ppm 22.73 (s, 2C) 26.74 (s, 1C) 28.36 (s, 3C) 29.65 (s, 2C) 41.47 (s, 2C) 52.61 (s, 2C) 62.74 (s, 1C) 80.42 (s, 1C) 110.55 (s, 1C) 114.49 (s, 1C) 124.93 (s, 1C) 127.80 (s, 2C) 128.07 (s, 4C) 129.52 (s, 5C) 129.92 (s, 3C) 133.12 (s, 2C) 137.81 (s, 1C) 153.94 (s, 1C) 155.94 (s, 2C) 157.51 (s, 1C); **HPLC-MS (ESI):** [R$_t$]: 3.88 min, *m/z* for $C_{38}H_{44}N_6O_4$ ([M+H$^+$]), 649.81 calcd., 649.40 found.

Yield (12b): 26 %. **^{1}H-NMR** (600 MHz, Chloroform-*d*) δ ppm 1.03 (d, *J* = 6.60 Hz, 6H) 1.49 (s, 9 H) 1.55 (s, 1H) 1.77 (s, 2H) 2.02 - 2.07 (m, 2H) 2.11 - 2.26 (m, 1H) 2.27 - 2.36 (m, 2H) 2.42 - 2.64 (m, 2H) 2.78 (d, *J* = 6.97 Hz, 2H) 2.96 - 3.20 (m, 2H) 3.69 - 3.76 (m, 1H) 6.94 (d, *J* = 8.25 Hz, 2H) 7.05 (s, 1H) 7.27 - 7.41 (m, 10H) 10.37 - 10.50 (m, 1H); **^{13}C-NMR** (151 MHz, Chloroform-*d*) δ ppm 22.69 (s, 2C) 26.76 (s, 1C) 28.37 (s, 3C) 29.62 (s, 2C) 32.05 (s, 1C) 33.96 (s, 1C) 41.56 (s, 1C) 52.98 (s, 1C) 58.54 (s, 1C) 80.66 (s, 1C) 109.72 (s, 1C) 110.15 (s, 1C) 113.00 (s, 1C) 114.27 (s, 1C) 123.94 (s, 1C) 128.70 (s, 2C) 129.04 (s, 2C) 129.45 (s, 4C) 129.75 (s, 2C) 130.38 (s, 1C) 133.08 (s, 1C) 134.35 (s, 1C) 135.07 (s, 1C) 139.49 (s, 1C) 153.60 (s, 1C) 155.71 (s, 1C) 156.76 (s, 1C) 157.83 (s, 1C); **HPLC-MS (ESI):** [R$_t$]: 4.23 min, *m/z* for $C_{38}H_{44}N_6O_4$ ([M+H$^+$]), 649.81 calcd., 649.39 found.

Synthesis of tert-butyl (3-(1-(4-(6-methyl-5-oxo-3-phenyl-4,5-dihydropyrazin-2-yl)benzyl)piperidin-4-yl)-2-oxo-2,3-dihydro-1H-benzo[d]imidazol-5-yl)carbamate (**11a**) *and tert-butyl (3-(1-(4-(5-methyl-6-oxo-3-phenyl-1,6-dihydropyrazin-2-yl)benzyl)piperidin-4-yl)-2-oxo-2,3-dihydro-1H-benzo[d]imidazol-5-yl)carbamate* (**12a**). *Tert*-butyl (2-oxo-3-(1-(4-(2-oxo-2-phenylacetyl)benzyl)piperidin-4-yl)-2,3-dihydro-1H-benzo[*d*]imidazol-5-yl)carbamate (**10**, 100 mg, 0.18 mmol, 1.0 eq.) and alanine carboxamide (88,1 mg, 0,54 mmol, 3.0 eq.) were used following common procedure A and yielded a mixture of the titled compounds as pale yellow solid (46 %). **^{1}H-NMR** (500 MHz, DMSO-*d$_6$*) δ ppm 1.48 (s, 9 H) 1.58 - 1.66 (m, 2H) 2.04 - 2.16 (m, 2H) 2.24 - 2.34 (m, 2H) 2.39 (s, 3H) 2.86 - 2.96 (m, 2H) 3.16 (s, 2H) 4.05 - 4.10 (m, 1H) 6.83 (d, *J* = 8.07 Hz, 1H) 7.03 (s, 1H) 7.12 - 7.22 (m, 4H) 7.24 - 7.28 (m, 1H) 7.29 - 7.38 (m, 3H) 7.46 (s, 1H) 7.58 - 7.73 (m, 1H) 9.15 (s, 1H) 10.68 (s, 1H) 12.20 (s, 1H); **^{13}C-NMR** (126 MHz, DMSO-*d$_6$*) δ ppm 28.18 (s, 3C) 28.48 (s, 2C) 48.57 (s, 1C) 49.92 (s, 1C) 52.50 (s, 2C) 61.29 (s, 1C) 78.66 (s, 1C) 108.51 (s, 2C) 111.11 (s, 1C) 123.40 (s, 2C) 126.86 (s, 1C) 127.69 (s, 2C) 128.25 (s, 2C) 128.56 (s, 1C) 129.02 (s, 2C) 129.19 (s, 2C) 129.65 (s, 3 C) 133.06 (s, 2C) 152.92 (s, 1C) 153.96 (s, 1C) 155.66 (s, 1C); **HPLC-MS (ESI): 11a** [R$_t$]: 3.38 min, *m/z* for $C_{35}H_{38}N_6O_2$ ([M+H$^+$]), 607.72 calcd., 607.36 found; **HPLC-MS (ESI): 12a** [R$_t$]: 3.61 min, *m/z* for $C_{35}H_{38}N_6O_2$ ([M+H$^+$]), 607.72 calcd., 607.35 found.

Synthesis of tert-butyl (3-(1-(4-(5-(4-hydroxybenzyl)-6-oxo-3-phenyl-1,6-dihydropyrazin-2-yl)benzyl)piperidin-4-yl)-2-oxo-2,3-dihydro-1H-benzo[d]imidazol-5-yl)carbamate (**12c**) *and tert-butyl (3-(1-(4-(6-(4-hydroxybenzyl)-5-oxo-3-phenyl-4,5-dihydropyrazin-2-yl)benzyl)piperidin-4-yl)-2-oxo-2,3-dihydro-1H-benzo[d]imidazol-5-yl)carbamate* (**11c**). *Tert*-butyl (2-oxo-3-(1-(4-(2-oxo-2-phenylacetyl)benzyl)piperidin-4-yl)-2,3-dihydro-1H-benzo[*d*]imidazol-5-yl)carbamate (**10**, 100 mg, 0.18 mmol, 1.0 eq.) and tyrosine amide (96.9 mg, 0.54 mmol, 3.0 eq.) were used following common procedure A and yielded a mixture of the titled compounds as a pale yellow solid (48 %). **^{1}H-NMR** (500 MHz, DMSO-*d$_6$*) δ ppm 1.46 (d, *J* = 5.80 Hz, 9 H) 1.57 - 1.66 (m, 2H) 2.02 - 2.14 (m, 2H) 2.22 - 2.34 (m, 2H) 2.92 (d, *J* = 8.70 Hz, 2H) 3.47 - 3.58 (m, 2H) 3,95 (s, 2H) 4.04 - 4.13 (m, 1H) 6.64 - 6.73 (m, 2H) 6.83 (d, *J* = 8.24 Hz, 1H) 6.98 - 7.06 (m, 1H) 7.09 - 7.22 (m, 5H) 7.23 - 7.39 (m, 6H) 7.47 (s, 1H) 9.15 - 9.25 (m, 2H) 10.70 (s, 1H); **^{13}C-NMR** (126 MHz, DMSO-*d$_6$*) δ ppm 28.22 (s, 3C) 28.52 (s, 2C) 37.70 (s, 1C) 49.91 (s, 1C) 52.56 (s, 2C) 61.27 (s, 1C) 78.77 (s, 1C) 108.59 (s, 1C) 109.69 (s, 1C) 114.83 (s, 1C) 115.09 (s, 4C) 123.43 (s, 2C) 126.97 (s, 1C) 127.79 (s, 2C) 128.19 (s, 1C) 128.35 (s, 2C) 128.67 (s, 1C) 129.02 (s, 1C) 129.24 (s, 2C) 129.62 (s, 1C) 129.72 (s, 2C) 130.03 (s, 3C) 133.14 (s, 2C) 152.98 (s, 1C) 154.01 (s, 1C) 155.83 (s, 1C); **HPLC-MS (ESI): 11c** [R$_t$]: 3.65 min, *m/z* for $C_{41}H_{42}N_6O_5$ ([M+H$^+$]), 699.82 calcd., 699.33 found; **HPLC-MS (ESI): 12c** [Rt]: 3.87 min, *m/z* for $C_{41}H_{42}N_6O_5$ ([M+H$^+$]), 699.82 calcd., 699.39 found.

*Synthesis of 6-amino-1-(1-(4-(5-isobutyl-6-oxo-3-phenyl-1,6-dihydropyrazin-2-yl)benzyl)piperidin-4-yl)-1,3-dihydro-2H-benzo[d]imidazol-2-one (**14b**).* *Tert*-butyl (3-(1-(4-(5-isobutyl-6-oxo-3-phenyl-4,5-dihydropyrazin-2-yl)benzyl)piperidin-4-yl)-2-oxo-2,3-dihydro-1*H*-benzo[*d*]imidazol-5-yl)carbamate (**12b**, 50.0 mg, 0.07 mmol, 1.0 eq.) and was used following common procedure B and yielded the titled compound as a pale yellow solid (13.1 mg, 0.02 mmol, 26 %). **^{1}H-NMR** (600 MHz, Methanol-*d$_4$*) δ ppm 1.02 (d, *J* = 6.60 Hz, 6H) 1.18 (t, *J* = 6.97 Hz, 1H) 1.73 (d, *J* = 10.82 Hz, 2H) 2.26 (d, *J* = 11.37 Hz, 2H) 2.50 (d, *J* =12.29 Hz, 2H) 2.73 (d, *J* = 7.15 Hz, 2H) 3.07 (d, *J* = 11.19 Hz, 2H) 3.58 - 3.64 (m, 1H) 3.65 (s, 2H) 4.21 - 4.30 (m, 1H) 6.50 (d, *J* = 9.72 Hz, 1H) 6.79 - 6.85 (m, 2H) 7.20 (m, 3H) 7.22 - 7.26 (m, 2H) 7.28 - 7.32 (m, 2H) 7.38 (s, 2H) 10.35 (s, 1H); **^{13}C-NMR** (151 MHz, Methanol-*d$_4$*) δ ppm 23.20 (s, 2C) 28.28 (s, 1C) 29.55 (s, 2C) 42.59 (s, 1C) 51.77 (s, 1C) 54.22 (s, 2C) 63.27 (s, 1C) 99.30 (s, 1C) 110.96 (s, 1C) 111.20 (s, 1C) 119.18 (s, 1C) 122.41 (s, 1C) 128.59 (s, 1C) 129.11 (s, 3C) 130.89 (s, 3C) 131.08 (s, 3C) 133.99 (s, 1C) 134.34 (s, 1C) 136.84 (s, 1C) 139.07 (s, 1C) 139.91 (s, 1C) 143.41 (s, 1C) 156.59 (s, 1C) 158.07 (s, 1C); **HRMS (ESI):** *m/z* for $C_{33}H_{37}N_6O_2$ ([M+H$^+$]), 549.2972 calcd., 549.2964 found.

Synthesis of 6-amino-1-(1-(4-(6-isobutyl-5-oxo-3-phenyl-4,5-dihydropyrazin-2-yl)benzyl)piperidin-4-yl)-1,3-dihydro-2H-benzo[d]imidazol-2-one (**13b**)*.* *Tert*-butyl (3-(1-(4-(6-isobutyl-5-oxo-3-phenyl-4,5-dihydropyrazin-2-yl)benzyl)piperidin-4-yl)-2-oxo-2,3-dihydro-1*H*-benzo[*d*]imidazol-5-yl)carbamate (**11b**, 48.0 mg, 0.07 mmol, 1.0 eq.) and was used following common procedure B and yielded the titled compound as a pale yellow solid (8.52 mg, 0.01 mmol, 21 %). **^{1}H-NMR** (600 MHz, DMSO-*d$_6$*) δ ppm 0.97 (d, *J* = 6.60 Hz, 6H) 1.59 (d, *J* = 10.09 Hz, 2H) 2.05 (s, 2H) 2.18 - 2.24 (m, 1H) 2.29 (d, *J* = 10.82 Hz, 2H) 2.64 (d, *J* = 6.97 Hz, 2H) 2.92 (d, *J*

= 6.79 Hz, 2H) 3.17 (d, *J* = 3.85 Hz, 2H) 4.01 - 4.16 (m, 1H) 4.74 (s, 2H) 6.21 (d, *J* = 7.89 Hz, 1H) 6.54 (s, 1H) 6.63 (d, *J* = 8.25 Hz, 1H) 7.12 - 7.22 (m, 4H) 7.28 - 7.42 (m, 5H) 10.29 (s, 1H) 12.19 (s, 1H);**^{13}C-NMR** (151 MHz, DMSO-*d$_6$*) δ ppm 23.02 (s, 2C) 26.69 (s, 2C) 29.00 (s, 1C) 41.33 (s, 1C) 49.01 (s, 2C) 53.10 (s, 1C) 61.86 (s, 1C) 96.27 (s, 1C) 107.16 (s, 1C) 109.51 (s, 1C) 119.49 (s, 2C) 128.71 (s, 4C) 129.42 (s, 3C) 130.10 (s, 4C) 130.28 (s, 2C) 137.12 (s, 1C) 143.49 (s, 2C) 154.34 (s, 1C) 156.07 (s, 1C); **HRMS (ESI):** *m/z* for $C_{33}H_{37}N_6O_2$ ([M+H$^+$]), 549.2972 calcd., 549.2967 found.

Synthesis of 6-amino-1-(1-(4-(5-methyl-6-oxo-3-phenyl-1,6-dihydropyrazin-2-yl)benzyl)piperidin-4-yl)-1,3-dihydro-2H-benzo[d]imidazol-2-on (**13a**). *Tert*-butyl (3-(1-(4-(5-methyl-6-oxo-3-phenyl-4,5-dihydropyrazin-2-yl)benzyl)piperidin-4-yl)-2-oxo-2,3-dihydro-1*H*-benzo[*d*]imidazol-5-yl)carbamate (**11a**, 52.0 mg, 0.09 mmol, 1.0 eq.) and was used following common procedure B and yielded the titled compound as a pale yellow solid (12.1 mg, 0.02 mmol, 28 %). **^{1}H-NMR** (500 MHz, DMSO-*d$_6$*) **δ** ppm 1.60 - 1.68 (m, 2H) 2.01 - 2.11 (m, 2H) 2.23 - 2.32 (m, 2H) 2.38 (s, 3H) 2.89 - 2.94 (m, 2H) 3.52 (s, 2H) 4.08 - 4.16 (m, 1H) 4.76 (s, 2H) 6.22 (d, *J* = 6.26 Hz, 1H) 6.54 (br. s., 1H) 6.64 (d, *J* = 8.54 Hz, 1H) 7.10 - 7.10 (m, 1H) 7.14 - 7.21 (m, 4H) 7.24 - 7.39 (m, 5H) 10.31 (br. s., 1H); **^{13}C-NMR** (126 MHz, DMSO-*d$_6$*) **δ** ppm 28.63 (s, 2C) 49.73 (s, 1C) 52.73 (s, 2C) 56.04 (s, 1C) 61.49 (s, 1C) 95.85 (s, 1C) 106.74 (s, 1C) 109.13 (s, 1C) 119.05 (s, 1C) 126.93 (s, 1C) 127.76 (s, 2C) 128.33 (s, 2C) 128.70 (s, 1C) 129.00 (s, 1C) 129.11 (s, 1C) 129.25 (s, 2C) 129.62 (s, 1C) 129.71 (s, 1C) 129.87 (s, 1C) 131.57 (s, 1C) 137.91 (s, 1C) 139.38 (s, 1C) 143.14 (s, 1C) 153.94 (s, 1C) 155.73 (s, 1C); **HRMS (ESI):** *m/z* for $C_{30}H_{31}N_6O_2$ ([M+H$^+$]), 507.2503 calcd., 507.2498 found.

Synthesis of 6-amino-1-(1-(4-(5-methyl-6-oxo-3-phenyl-1,6-dihydropyrazin-2-yl)benzyl)piperidin-4-yl)-1,3-dihydro-2H-benzo[d]imidazol-2-one (**14a**). *Tert*-butyl (3-(1-(4-(5-methyl-6-oxo-3-phenyl-4,5-dihydropyrazin-2-yl)benzyl)piperidin-4-yl)-2-oxo-2,3-dihydro-1*H*-benzo[*d*]imidazol-5-yl)carbamate (**12a**, 52.0 mg, 0.09 mmol, 1.0 eq.) and was used following common procedure B and yielded the titled compound as a pale yellow solid (12.1 mg, 0.02 mmol, 28 %). **^{1}H-NMR** (500 MHz, DMSO-*d$_6$*) δ ppm 1.60 - 1.68 (m, 2H) 2.01 - 2.11 (m, 2H) 2.23 - 2.32 (m, 2H) 2.38 (s, 3H) 2.89 - 2.94 (m, 2H) 3.52 (s, 2H) 4.08 - 4.16 (m, 1H) 4.76 (s, 2H) 6.22 (d, *J* = 6.26 Hz, 1H) 6.54 (s, 1H) 6.64 (d, *J* = 8.54 Hz, 1H) 7.10 - 7.10 (m, 1H) 7.14 - 7.21 (m, 4H) 7.24 - 7.39 (m, 5H) 10.31 (s, 1H); **^{13}C-NMR** (126 MHz, DMSO-*d$_6$*) δ ppm 28.63 (s, 2C) 49.73 (s, 1C) 52.73 (s, 2C) 56.04 (s, 1C) 61.49 (s, 1C) 95.85 (s, 1C) 106.74 (s, 1C) 109.13 (s, 1C) 119.05 (s, 1C) 126.93 (s, 1C) 127.76 (s, 2C) 128.33 (s, 2C) 128.70 (s, 1C) 129.00 (s, 1C) 129.11 (s, 1C) 129.25 (s, 2C) 129.62 (s, 1C) 129.71 (s, 1C) 129.87 (s, 1C) 131.57 (s, 1C) 137.91 (s, 1C) 139.38 (s, 1C) 143.14 (s, 1C) 153.94 (s, 1C) 155.73 (s, 1C); **HRMS (ESI):** *m/z* for $C_{30}H_{31}N_6O_2$ ([M+H$^+$]), 507.2503 calcd., 507.2498 found.

Synthesis of 6-amino-1-(1-(4-(5-(4-hydroxybenzyl)-6-oxo-3-phenyl-1,6-dihydropyrazin-2-yl)benzyl)piperidin-4-yl)-1,3-dihydro-2H-benzo[d]imidazol-2-one (**14c**). *Tert*-butyl (3-(1-(4-(5-(4-hydroxybenzyl)-6-oxo-3-phenyl-1,6-dihydropyrazin-2-yl)benzyl)piperidin-4-yl)-2-oxo-2,3-dihydro-1*H*-benzo[*d*]imidazol-5-yl)carbamate (**12c**, 62.0 mg, 0.09 mmol, 1.0 eq.) and was used following common procedure B and yielded the titled compound as a pale yellow solid (10.6 mg, 0.01 mmol, 20 %). **^{1}H-NMR** (700 MHz, DMSO-*d$_6$*) δ d ppm 1.60 (d, *J* = 8.60 Hz, 2H) 1.95 - 2.13 (m, 2H) 2.20 - 2.36 (m, 2H) 2.83 - 3.00 (m, 2H) 3.47 - 3.59 (m, 2H) 3.95 (s, 2H) 4.00 - 4.13 (m, 1H) 4.73 (s, 2H) 6.21 (d, *J* = 7.96 Hz, 1H) 6.51 - 6.64 (m, 2H) 6.65 - 6.72 (m, 2H) 6.92 (m, 1H) 7.11 - 7.23 (m, 5H) 7.23 - 7.34 (m, 5H) 9.20 (s, 1H) 10.29 (s, 1H); **^{13}C-NMR** (176 MHz, DMSO-*d$_6$*) δ ppm 28.59 (s, 2C) 37.63 (s, 2C) 49.73 (s, 1C) 52.68 (s, 2C) 61.50 (s, 1C) 95.83 (s, 1C) 106.75 (s, 1C) 109.09 (s, 1C) 114.67 (s, 1C) 115.04 (s, 2C) 119.06 (s, 1C) 126.93 (s, 1C) 127.74 (s, 3C) 128.09 (s, 1C) 128.69 (s, 1C) 129.23 (s, 3C) 129.57 (s, 1C) 129.85 (s, 1C) 129.96 (s, 3C) 132.58 (s, 1C) 137.81 (s, 1C) 143.06 (s, 1C) 153.91 (s, 1C) 155.27 (s, 1C) 155.79 (s, 1C) 156.54 (s, 1C); **HRMS (ESI):** *m/z* for $C_{36}H_{35}N_6O_3$ ([M+H$^+$]), 599.2765 calcd., 599.2764 found.

Synthesis of 6-amino-1-(1-(4-(6-(4-hydroxybenzyl)-5-oxo-3-phenyl-4,5-dihydropyrazin-2-yl)benzyl)piperidin-4-yl)-1,3-dihydro-2H-benzo[d]imidazol-2-one (**13c**). *Tert*-butyl (3-(1-(4-(6-(4-hydroxybenzyl)-5-oxo-3-phenyl-1,6-dihydropyrazin-2-yl)benzyl)piperidin-4-yl)-2-oxo-2,3-dihydro-1*H*-benzo[*d*]imidazol-5-yl)carbamate (**11c**, 62.0 mg, 0.09 mmol, 1.0 eq.) and was used following common procedure B and yielded the titled compound as a pale yellow solid (11.6 mg, 0.01 mmol, 22 %). **^{1}H-NMR** (600 MHz, DMSO-*d$_6$*) δ ppm 1.51 - 1.68 (m, 2H) 1.97 - 2.15 (m, 2H) 2.21 - 2.35 (m, 2H) 2.85 - 2.99 (m, 2H) 3.48 - 3.60 (m, 2H) 3.95 (s, 2H) 4.00 - 4.12 (m, 1H) 4.60 - 4.86 (m, 1H) 6.19 - 6.24 (m, 1H) 6.54 (s, 1H) 6.63 (d, *J* = 8.07 Hz, 1H) 6.68 (d, *J* = 7.15 Hz, 2H) 7.09 - 7.23 (m, 7H) 7.23 - 7.41 (m, 6H) 9.19 (s, 1H) 10.29 (s, 1H); **^{13}C-NMR** (151 MHz, DMSO-*d$_6$*) δ ppm 28.59 (s, 2C) 37.63 (s, 1C) 49.75 (s, 1C) 52.66 (s, 2C) 56.04 (s, 1C) 61.51 (s, 1C) 95.84 (s, 1C) 106.73 (s, 1C) 109.05 (s, 1C) 115.03 (s, 2C) 119.09 (s, 1C) 126.92 (s, 1C) 127.72 (s, 3C) 128.07 (s, 2C) 128.27 (s, 2C) 129.20 (s, 3C) 129.55 (s, 2C) 129.85 (s, 2C) 129.98 (s, 3C) 143.04 (s, 1C) 153.89 (s, 1C) 155.77 (s, 2C); **HRMS (ESI):** *m/z* for $C_{36}H_{35}N_6O_3$ ([M+H$^+$]), 599.2765 calcd., 599.2765 found.

Synthesis of N-(3-(1-(4-(5-isobutyl-6-oxo-3-phenyl-1,6-dihydropyrazin-2-yl)benzyl)piperidin-4-yl)-2-oxo-2,3-dihydro-1H-benzo[d]imidazol-5-yl)acrylamide (**16b**). 6-Amino-1-(1-(4-(5-isobutyl-6-oxo-3-phenyl-1,6-dihydropyrazin-2-yl)benzyl)piperidin-4-yl)-1,3-dihydro-2*H*-benzo[*d*]imidazol-2-one (**14b**, 13.1 mg, 0.02 mmol, 1.0 eq.) and was used following common procedure C and yielded the titled compound as a pale yellow solid (12.9 mg, 0.02 mmol, 90 %). **^{1}H-NMR** (500 MHz, DMSO-*d$_6$*) δ ppm 0.96 (d, *J* = 6.56 Hz, 6H) 1.66 (d, *J* = 10.38 Hz, 2H) 2.09 (t, *J* = 11.37 Hz, 2H) 2.21 (quin, *J* = 6.70 Hz, 1H) 2.29 (q, *J* = 9.90 Hz, 2H) 2.63 (d, *J* = 7.02 Hz, 2H) 2.94 (d, *J* = 10.53 Hz, 2H) 3.55 (s, 2H) 4.07 - 4.15 (m, 1H) 5.73 (d, *J* = 10.99 Hz, 1H) 6.24 (d, *J* = 16.63 Hz, 1H) 6.41 (dd, *J* = 16.94, 10.07 Hz, 1H) 6.92 (d, *J* = 8.39 Hz, 1H) 7.19 (s, 5H) 7.25 - 7.34 (m, 5H) 7.72 (s, 1H) 10.10 (s, 1H) 10.83 (s, 1H); **^{13}C-NMR** (126 MHz, DMSO-*d$_6$*) δ ppm 22.64 (s, 2C) 26.28 (s, 1C) 28.69 (s, 2C) 40.95 (s, 1C) 50.11 (s, 1C) 52.64 (s, 2C) 56.05 (s, 1C) 61.49 (s, 1C) 100.92 (s, 1C) 108.70 (s, 1C) 112.12 (s, 1C) 124.50 (s, 1C) 126.42 (s, 1C) 126.92 (s, 1C) 127.79 (s, 3C) 128.83 (s, 2C) 128.97 (s, 1C) 129.21 (s, 3C) 129.64 (s, 2C) 132.05 (s, 1C) 132.68 (s, 1C) 138.01 (s, 1C) 139.24 (s, 1C) 154.02 (s, 2C) 155.67 (s, 1C) 162.79 (s, 1C); **HRMS (ESI):** *m/z* for $C_{36}H_{39}N_6O_3$ ([M+H$^+$]), 603.3078 calcd., 603.3075 found.

Synthesis of N-(3-(1-(4-(6-Isobutyl-5-oxo-3-phenyl-4,5-dihydropyrazin-2-yl)benzyl)piperidin-4-yl)-2-oxo-2,3-dihydro-1H-benzo[d]imidazol-5-yl)acrylamide (**15b**). 6-Amino-1-(1-(4-(6-isobutyl-5-oxo-3-phenyl-1,6-dihydropyrazin-2-yl)benzyl)piperidin-4-yl)-1,3-dihydro-2*H*-benzo[*d*]imidazol-2-one (**13b**, 8.52 mg, 0.01 mmol, 1.0 eq.) and was used following common procedure C and yielded the titled compound as a pale yellow solid (6.64 mg, 0.091 mmol, 71 %). **^{1}H-NMR** (500 MHz, DMSO-d_6) δ ppm 0.96 (d, *J* = 6.71 Hz, 6H) 1.64 (d, *J* = 9.92 Hz, 2H) 2.02 - 2.11 (m, 2H) 2.22 (dd, *J* = 13.66, 6.79 Hz, 1H) 2.28 (d, *J* = 11.60 Hz, 2H) 2.64 (d, *J* = 6.87 Hz, 2H) 2.88 - 2.96 (m, 2H) 3.48 (s, 2H) 4.05 - 4.14 (m, 1H) 5.73 (dd, *J* = 10.15, 1.75 Hz, 1H) 6.21 - 6.27 (m, 1H) 6.39 (s, 1H) 6.91 (d, *J* = 8.39 Hz, 1H) 7.13 - 7.22 (m, 4H) 7.25 - 7.41 (m, 7H) 7.69 (s, 1H) 10.09 (s, 1H) 10.82 (s, 1H); **^{13}C-NMR** (126 MHz, DMSO-d_6) δ ppm 22.64 (s, 2C) 26.28 (s, 1C) 28.69 (s, 2C) 40.94 (s, 1C) 50.17 (s, 1C) 52.66 (s, 2C) 61.54 (s, 1C) 100.99 (s, 1C) 106.73 (s, 1C) 108.66 (s, 1C) 112.11 (s, 1C) 119.03 (s, 1C) 124.47 (s, 1C) 126.42 (s, 2C) 128.34 (s, 4C) 128.95 (s, 3C) 129.75 (s, 3C) 132.05 (s, 2C) 132.58 (s, 2C) 143.13 (s, 1C) 153.96 (s, 1C) 155.72 (s, 1C) 162.78 (s, 1C); **HRMS (ESI):** *m/z* for $C_{36}H_{39}N_6O_3$ ([M+H$^+$]), 603.3078 calcd., 603.3067 found.

Synthesis of N-(3-(1-(4-(6-methyl-5-oxo-3-phenyl-4,5-dihydropyrazin-2-yl)benzyl)piperidin-4-yl)-2-oxo-2,3-dihydro-1H-benzo[d]imidazol-5-yl)acrylamide (**15a**). 6-Amino-1-(1-(4-(6-methyl-5-oxo-3-phenyl-1,6-dihydropyrazin-2-yl)benzyl)piperidin-4-yl)-1,3-dihydro-2*H*-benzo[*d*]imidazol-2-one (**13a**, 21.0 mg, 0.04 mmol, 1.0 eq.) and was used following common procedure C and yielded the titled compound as a pale yellow solid (5.08 mg, 0.01 mmol, 23 %). **^{1}H-NMR** (500 MHz, DMSO-d_6) δ ppm 1.56 - 1.70 (m, 2H) 1.98 - 2.13 (m, 2H) 2.21 - 2.32 (m, 2H) 2.38 (s, 3H) 2.87 - 3.00 (m, 2H) 3.44 - 3.54 (m, 2H) 4.05 - 4.15 (m, 1H) 5.74 (d, *J* = 11.29 Hz, 1H) 6.24 (d, *J* = 16.02 Hz, 1H) 6.41 (q, *J* = 9.00 Hz, 1H) 6.91 (d, *J* = 8.24 Hz, 1H) 7.13 - 7.22 (m, 4H) 7.25 - 7.42 (m, 7H) 7.65 - 7.73 (m, 1H) 10.09 (s, 1H) 10.82 (s, 1H); **^{13}C-NMR** (126 MHz, DMSO-d_6) δ ppm 28.63 (s, 2C) 29.12 (s, 1C) 50.12 (s, 1C) 52.66 (s, 2C) 61.50 (s, 1C) 100.95 (s, 1C) 108.69 (s, 2C) 124.50 (s, 2C) 126.42 (s, 2C) 128.35 (s, 4C) 128.98 (s, 1C) 129.16 (s, 2C) 129.73 (s, 4C) 132.05 (s, 2C) 132.66 (s, 2C) 154.01 (s, 2C) 155.78 (s, 1C) 162.78 (s, 1C); **HRMS (ESI):** *m/z* for $C_{33}H_{33}N_6O_3$ ([M+H$^+$]), 561.2608 calcd., 561.5979 found.

Synthesis of N-(3-(1-(4-(5-methyl-6-oxo-3-phenyl-1,6-dihydropyrazin-2-yl)benzyl)piperidin-4-yl)-2-oxo-2,3-dihydro-1H-benzo[d]imidazol-5-yl)acrylamide (**16a**). 6-Amino-1-(1-(4-(5-methyl-6-oxo-3-phenyl-1,6-dihydropyrazin-2-yl)benzyl)piperidin-4-yl)-1,3-dihydro-2*H*-benzo[*d*]imidazol-2-one (**14a**, 27.5 mg, 0.05 mmol, 1.0 eq.) and was used following common procedure C and yielded the titled compound as a pale yellow solid (5.15 mg, 0.01 mmol, 17 %). **^{1}H-NMR** (500 MHz, DMSO-d_6) δ ppm 1.59 - 1.72 (m, 2H) 2.04 - 2.18 (m, 2H) 2.23 - 2.34 (m, 2H) 2.38 (s, 3H) 2.90 - 2.99 (m, 2H) 3.56 (br. s., 2H) 4.07 - 4.16 (m, 1H) 5.73 (d, *J* = 10.53 Hz, 1H) 6.23 (d, *J* = 16.94 Hz, 1H) 6.41 (q, *J* = 10.10 Hz, 1H) 6.92 (d, *J* = 8.24 Hz, 1H) 7.15 - 7.22 (m, 5H) 7.23 - 7.38 (m, 5H) 7.72 (s, 2H) 10.11 (s, 1H) 10.83 (s, 1H); **^{13}C-NMR** (126 MHz, DMSO-d_6) δ ppm 28.23 (s, 2C) 29.04 (s, 1C) 50.09 (s, 1C) 52.60 (s, 2C) 61.45 (s, 1C) 100.93 (s, 1C) 108.70 (s, 1C) 112.14 (s, 2C) 124.49 (s, 2C) 126.41 (s, 2C) 126.94 (s, 1C) 127.78 (s, 3C) 128.96 (s, 2C) 129.26 (s, 3C) 129.66 (s, 2C) 132.05 (s, 2C) 132.67 (s, 1C) 154.01 (s, 2C) 154.14 (s, 1C) 162.80 (s, 1C); **HRMS (ESI):** *m/z* for $C_{33}H_{33}N_6O_3$ ([M+H$^+$]), 561.2608 calcd., 561.2590 found.

Synthesis of N-(3-(1-(4-(5-(4-hydroxybenzyl)-6-oxo-3-phenyl-1,6-dihydropyrazin-2-yl)benzyl)piperidin-4-yl)-2-oxo-2,3-dihydro-1H-benzo[d]imidazol-5-yl)acrylamide (**16c**). 6-Amino-1-(1-(4-(5-(4-hydroxybenzyl)-6-oxo-3-phenyl-1,6-dihydropyrazin-2-yl)benzyl)piperidin-4-yl)-1,3-dihydro-2*H*-benzo[*d*]imidazol-2-one (**14c**, 31.0 mg, 0.05 mmol, 1.0 eq.) and was used following common procedure C and yielded the titled compound as a pale yellow solid (11.1 mg, 0.01 mmol, 33 %). **^{1}H-NMR** (600 MHz, DMSO-d_6) δ ppm 1.57 - 1.73 (m, 2H) 2.03 - 2.15 (m, 2H) 2.23 - 2.35 (m, 2H) 2.87 - 3.00 (m, 2H) 3.50 - 3.59 (m, 2H) 3.95 (s, 2H) 4.06 - 4.15 (m, 1H) 5.74 (s, 1H) 6.19 - 6.28 (m, 1H) 6.37 - 6.47 (m, 1H) 6.68 (d, *J* = 8.44 Hz, 2H) 6.89 - 6.95 (m, 1H) 7.12 - 7.23 (m, 8 H) 7.24 - 7.37 (m, 6H) 9.20 (s, 1H) 10.07 - 10.14 (m, 1H) 10.76 - 10.86 (m, 1H); **^{13}C-NMR** (151 MHz, DMSO-d_6) δ ppm 28.58 (s, 2C) 41.69 (s, 1C) 50.11 (s, 1C) 52.59 (s, 1C) 53.42 (s, 2C) 61.44 (s, 1C) 100.94 (s, 1C) 108.68 (s, 1C) 112.14 (s, 1C) 115.03 (s, 5C) 124.46 (s, 1C) 126.34 (s, 2C) 126.97 (s, 1C) 127.79 (s, 2C) 128.92 (s, 2C) 129.25 (s, 2C) 129.58 (s, 1C) 130.00 (s, 4C) 131.98 (s, 2C) 132.64 (s, 1C) 154.01 (s, 2C) 155.80 (s, 2C) 162.80 (s, 1C) **HRMS (ESI):** *m/z* for $C_{39}H_{37}N_6O_3$ ([M+H$^+$]), 653.2870 calcd., 653.2869 found.

Synthesis of N-(3-(1-(4-(6-(4-hydroxybenzyl)-5-oxo-3-phenyl-4,5-dihydropyrazin-2-yl)benzyl)piperidin-4-yl)-2-oxo-2,3-dihydro-1H-benzo[d]imidazol-5-yl)acrylamide (**15c**). 6-Amino-1-(1-(4-(6-(4-hydroxybenzyl)-5-oxo-3-phenyl-1,6-dihydropyrazin-2-yl)benzyl)piperidin-4-yl)-1,3-dihydro-2H-benzo[d]imidazol-2-one (**13c**, 25.0 mg, 0.04 mmol, 1.0 eq.) and was used following common procedure C and yielded the titled compound as a pale yellow solid (10.9 mg, 0.01 mmol, 45 %). **^{1}H NMR** (500 MHz, DMSO-d_6) □ ppm 1.57 - 1.72 (m, 2H) 1.83 - 2.00 (m, 2H) 2.20 - 2.35 (m, 2H) 2.85 - 3.03 (m, 2H) 3.41 - 3.51 (m, 2H) 3.96 (s, 2H) 4.22 - 4.29 (m, 1H) 5.74 (d, J = 2.51 Hz, 1H) 6.24 (d, J = 16.78 Hz, 1H) 6.37 - 6.49 (m, 1H) 6.68 (d, J = 8.39 Hz, 2H) 6.87 - 6.96 (m, 1H) 7.11 - 7.21 (m, 4H) 7.33 (s, 5H) 7.59 - 7.89 (m, 3H) 8.17 - 8.43 (m, 2H) 9.23 (s, 1H) 10.11 (s, 1H) 10.82 - 10.92 (m, 1H); **^{13}C NMR** (126 MHz, DMSO-d_6) □ ppm 28.83 (s, 2C) 48.90 (s, 1C) 51.19 (s, 1C) 52.86 (s, 2C) 61.76 (s, 1C) 108.85 (s, 1C) 115.19 (s, 4C) 124.75 (s, 2C) 126.50 (s, 2C) 128.12 (s, 1C) 128.48 (s, 3C) 129.12 (s, 2C) 129.79 (s, 2C) 130.18 (s, 4C) 131.07 (s, 2C) 132.02 (s, 2C) 142.83 (s, 1C) 144.50 (s, 1C) 154.08 (s, 2C) 155.92 (s, 2C) 162.87 (s, 1C); **HPLC-MS (ESI):** [R_t]: 3.19 min, m/z for $C_{39}H_{37}N_6O_3$ ([M+H$^+$]), 653.28 calcd., 653.2883 found.

Results and Discussion

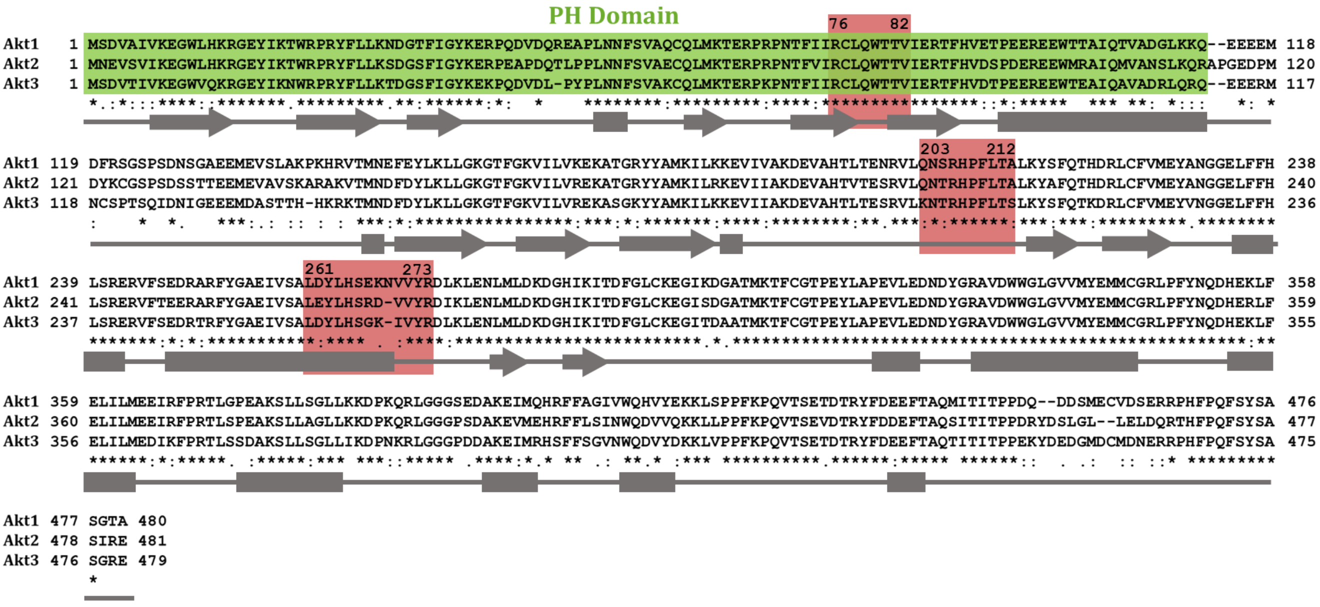

Figure S1. Sequence Alignment of the three Akt Isoforms. The Alignment was performed with Clustal Omega[1] and gave an overall identity of 73 %. The sequence of the PH Domain is coloured green and the secondary structure elements are indicated below the sequence (grey; tube – α-helix, arrow – β-sheet). Conserved amino acids in the sequence are marked below with (*), conservative mutations (:), semi-conservative mutations (.), and non-conservative mutations with ( ). Highlighted in red are the amino acids as well as secondary structure elements which form the inter domain binding pocket and presumably interact with the allosteric ligand.

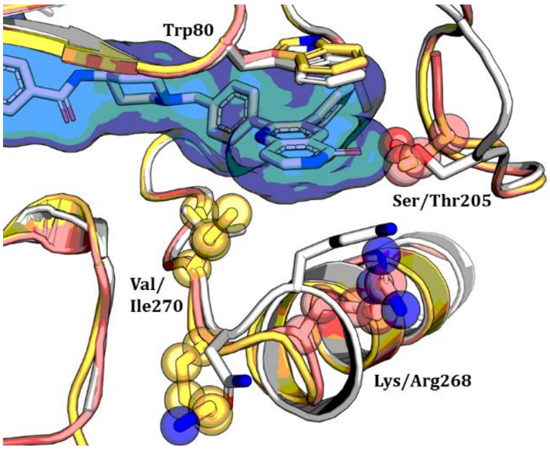

Figure S2. Homology model of Akt2 (red) and Akt3 (yellow) based on Akt1 complex crystal structure 6HHI (white) generated with SWISS-MODEL.[2] Altered residues which are in close contact to the ligand (blue) within the allosteric binding pocket, are labelled exclusively (residue numeration are based on the Akt1 complex template).

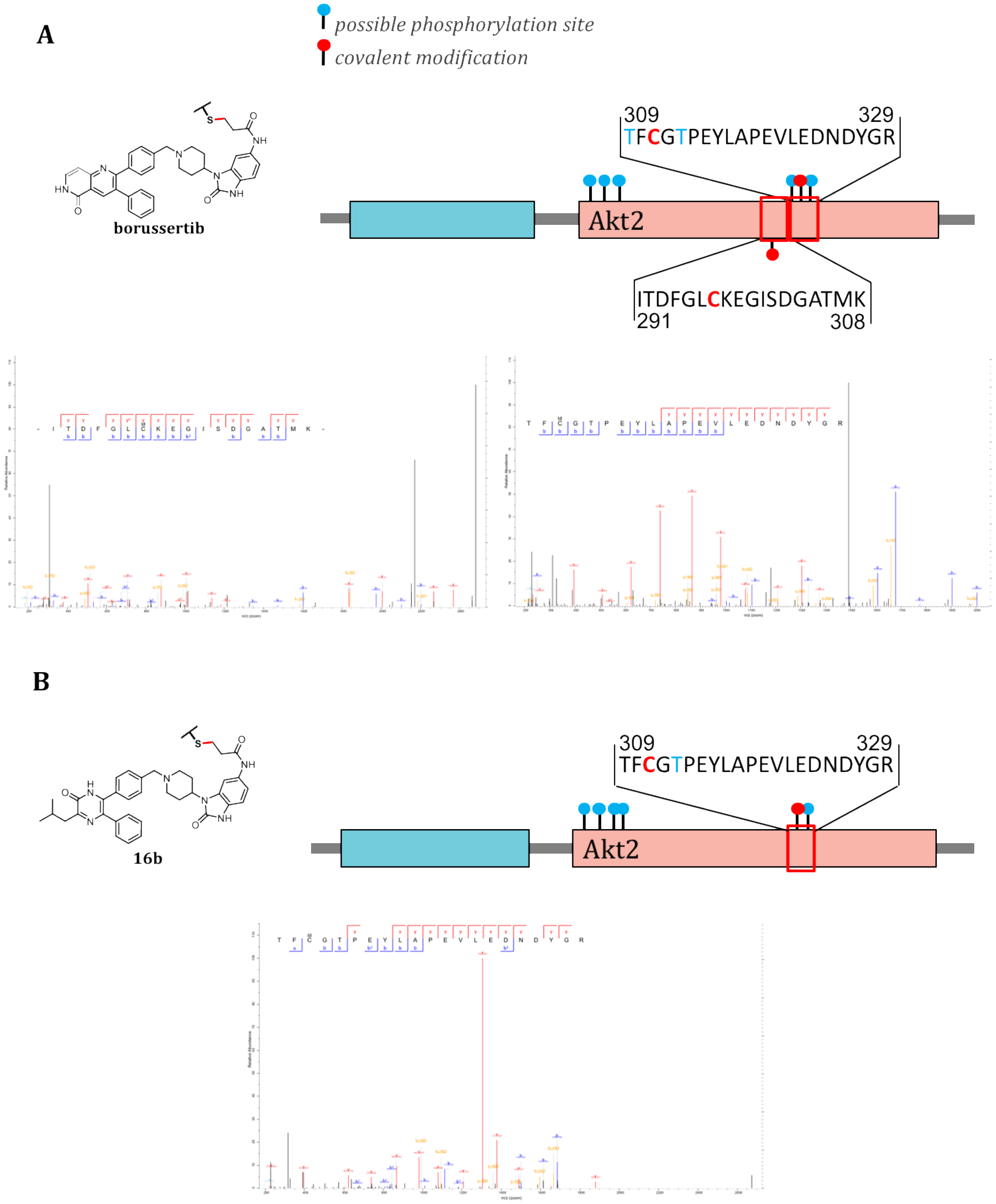

Figure S3. CAAI's covalently bind to Akt2 at Cys297 and Cys311, scheme of modifications and MS-spectra of peptide fragmentation pattern. Full-length Akt2 was incubated with a 5-fold molar excess of borussertib (**A**) and the novel isoform-selective inhibitor **16b** (**B**) and digested with trypsin after SDS-Page following standard protocol. Peptide fragments containing Cys297 and Cys311 modification with borussertib were identified, whereas for **16b,** only a selective labeling of Cys311 could be observed (sequence coverage >90 %).

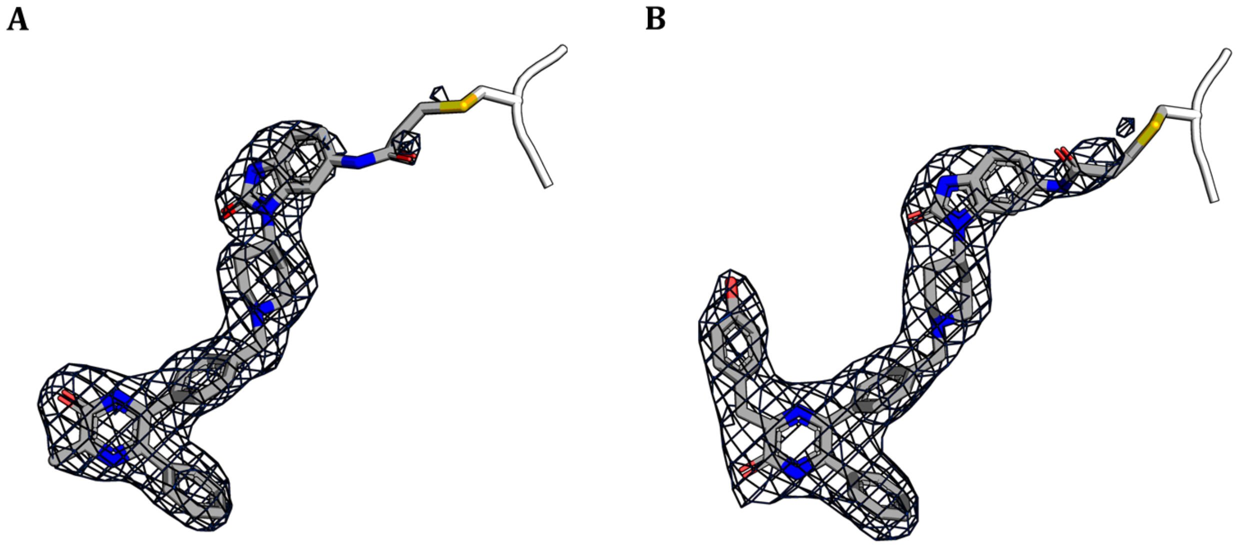

Figure S4. Performing a simulated annealing refinement, mFo-DFc omit maps (2.5σ) were calculated for **16a** (**A**) and **15c** (**B**).

A

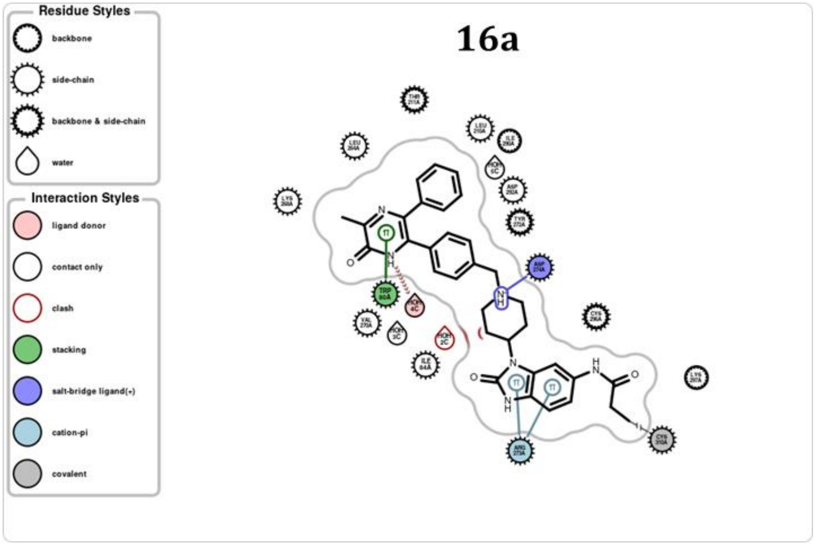

B

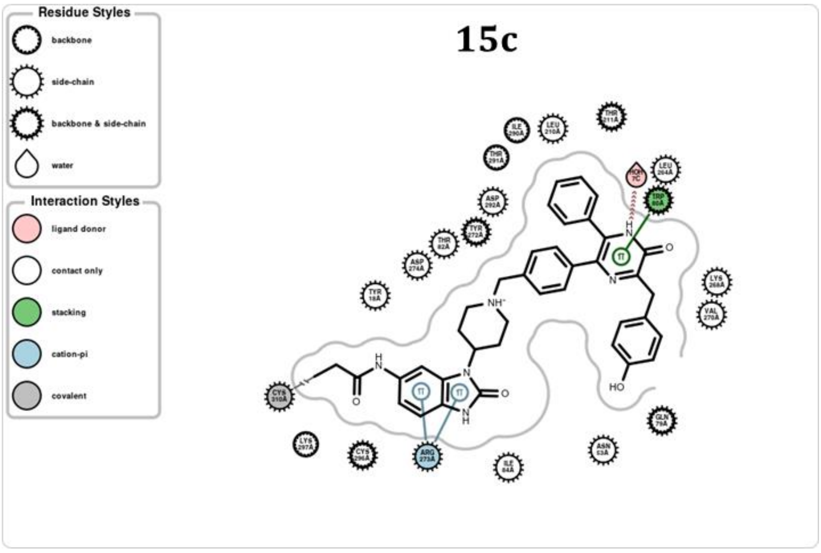

Figure S5. Close-up on the protein-ligand interactions of **16a** (**A**) and **15c** (**B**) based on the complex crystal structures illustrated the differences in the interaction profile of each ligand to the protein. The figures were generated by the OEChem/OEDepict toolkits from OpenEye Scientific Software (2019, Santa Fe, NM, www.eyesopen.com).

Table S1. Data collection and refinement statistics for full-length Akt1 in complex with different covalent-allosteric Akt inhibitors.

	**Akt1 with 16a**	**Akt1 with 15c**
**Data collection**	PDB: 6S9W	PDB: 6S9X
Space group	$P2_12_12_1$	$P2_12_12_1$
Cell dimensions		
a, b, c (Å)	70.07, 71.03, 91.34	71.46, 71.33, 91.56
α, β, γ (°)	90, 90, 90	90, 90, 90
Resolution (Å)	45.67 – 2.30 (2.40 – 2.30)	45.78 – 2.60 (2.70 – 2.60)
R_{meas} (%)	10.2 (170.5)	5.1 (156.6)
R_{merge} (%)	9.8 (163.7)	4.9 (150.8)
$I/\sigma I$	15.06 (1.62)	30.05 (1.8)
$CC_{1/2}$	99.9 (62.9)	100 (76.4)
Completeness (%)	91.8 (100)	100 (100)
Redundancy	12.6 (12.9)	13.0 (13.8)
**Refinement**		
Resolution (Å)	45.67 – 2.30	38.53 – 2.60
No. reflections	19149	14732
R_{work}/R_{free}	20.47/26.06 (28.65/36.85)	21.15/23.63 (35.99/38.18)
No. atoms		
Protein	3394	3162
Ligand/ion	42	49
Water	12	12
B-factors		
Protein	70.94	98.84
Ligand/ion	58.65	82.24
Water	54.54	87.74
rms deviations		
Bond lengths (Å)	0.003	0.003
Bond angles (°)	0.633	0.611
Wavelength (Å)	0.91886	0.91955
Temperature (K)	100	100
X-ray source	PX II at SLS, Villigen, CH	PX II at SLS, Villigen, CH
Detector	Pilatus 6MF	Pilatus 6MF
**Ramachandran Plot**		
Outliers (%)	0.00	0.00
Allowed (%)	1.98	2.42
Favored (%)	98.02	97.58

Table S2. Cellular Evaluation of covalent-allosteric Akt inhibitors with different cancer cell lines. Growth impairment was investigated with two breast cancer cell lines, ZR-75-1 and T47-D, bearing either PTEN loss-of-function or PI3K activating mutation, respectively, and the AN3CA cell line that incorporates both alterations.[19]

#	ZR-75-1 EC$_{50}$ [nM]			T47-D EC$_{50}$ [nM]			AN3CA EC$_{50}$ [nM]		
**capivasertib**	191	±	68	475	±	92	869	±	278
**MK-2206**	571	±	111	411	±	23	972	±	322
**borussertib**	5	±	1	48	±	15	191	±	90
**15a**	182	±	86	1146	±	608	7555	±	3913
**16a**	339	±	290	1448	±	432	19563	±	7381
**15b**	425	±	156	11735	±	5580	7089	±	722
**16b**	1738	±	450	2933	±	1159	5465	±	1270
**15c**	1890	±	678	9763	±	595	21462	±	6058
**16c**	1808	±	443	6610	±	586	17856	±	5378

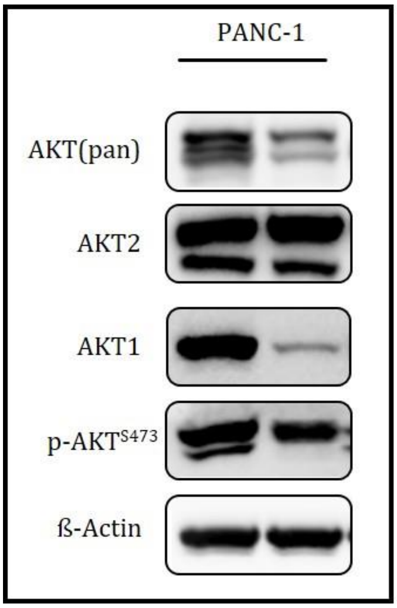

Figure S6. Western Blot analyses for cancer cell line PANC1 with Akt1 *knock-out*;(Left lane: untreated PANC-1 cells, right lane: CRISPR/Cas9 transduced Akt1 knock-out cells).

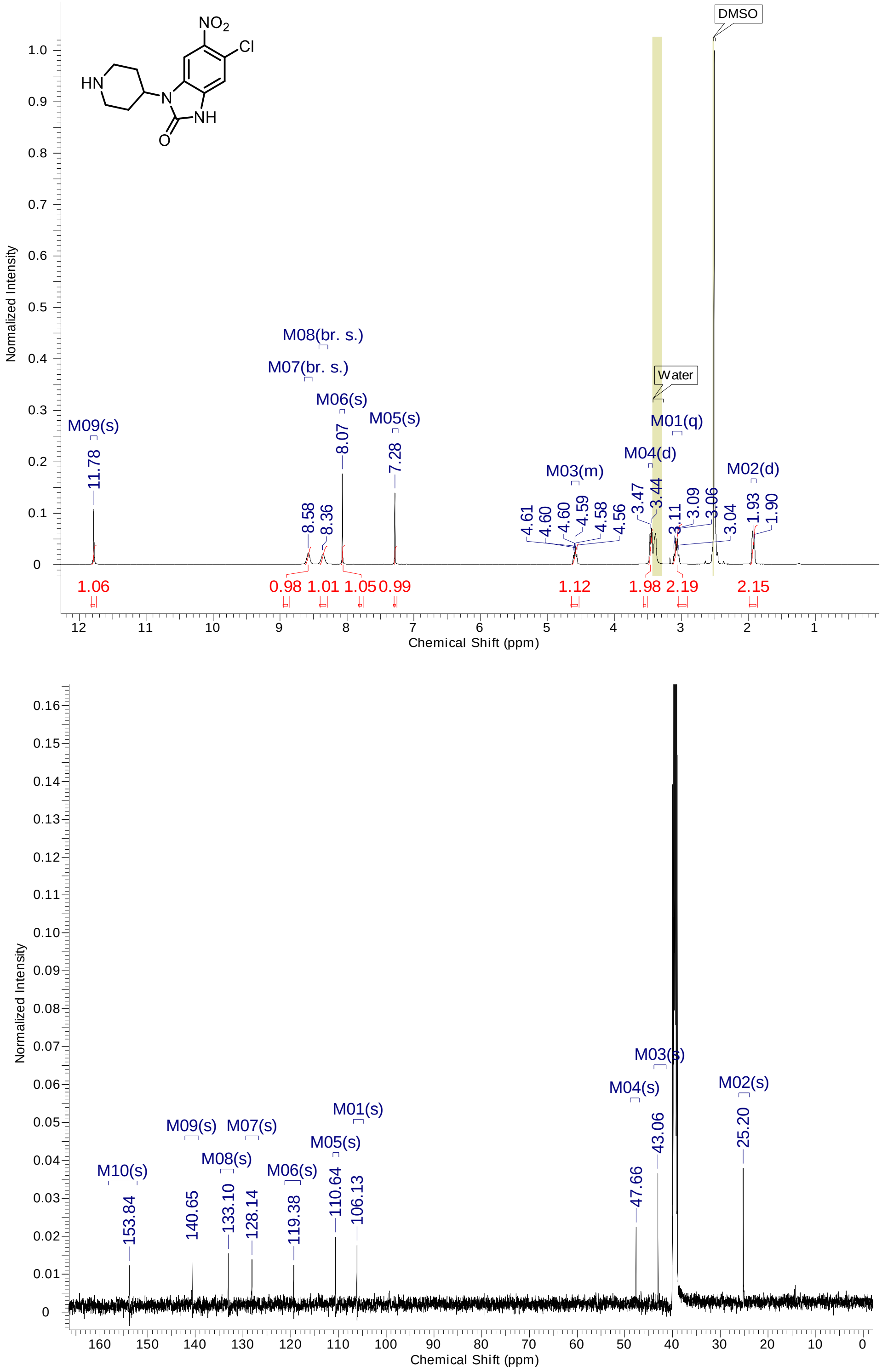

Figure S7. ^{1}H- and ^{13}C-NMR spectra of **2**.

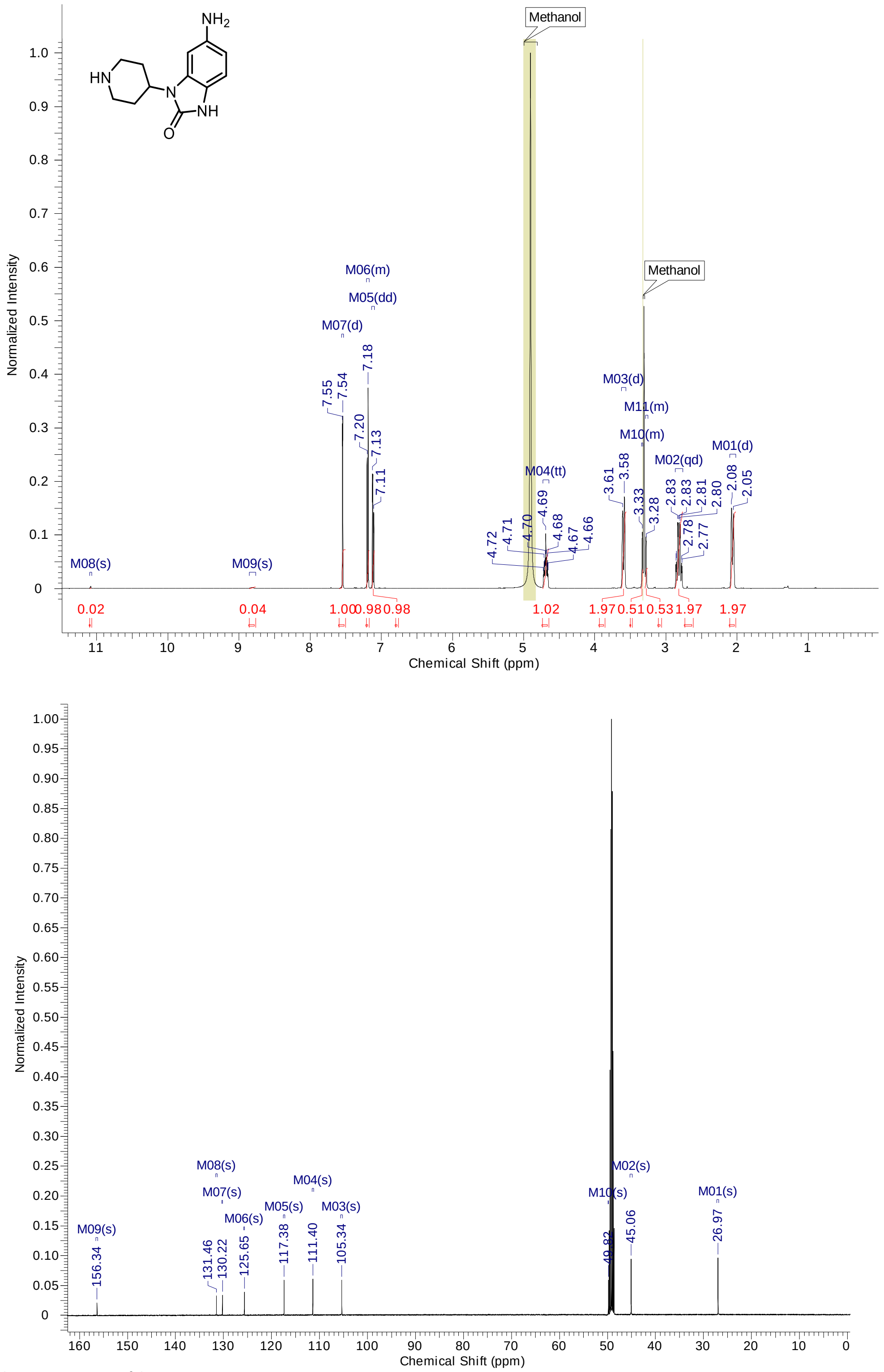

Figure S8. ^{1}H- and ^{13}C-NMR spectra of **3**.

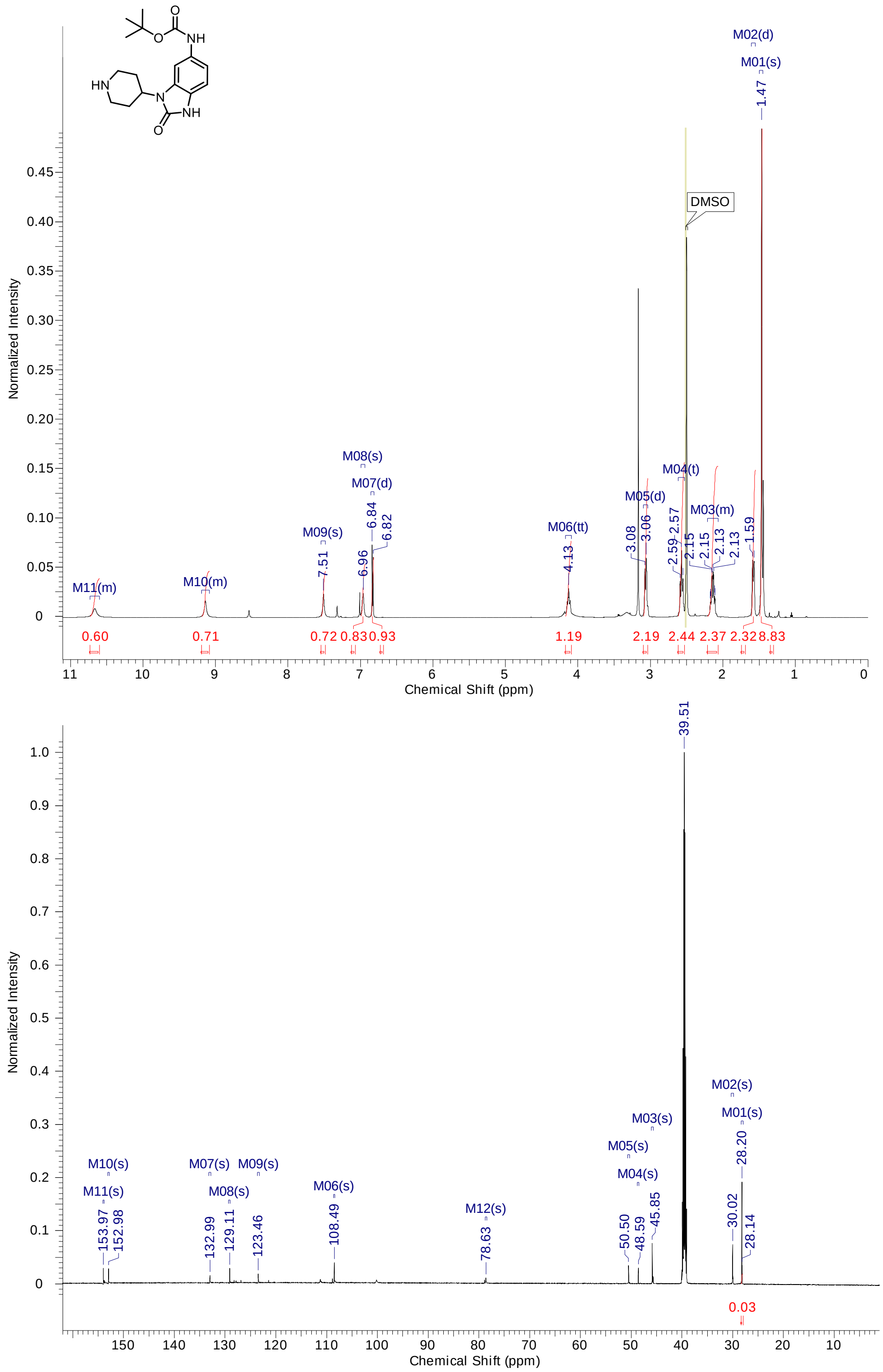

Figure S9. ^{1}H- and ^{13}C-NMR spectra of **4**.

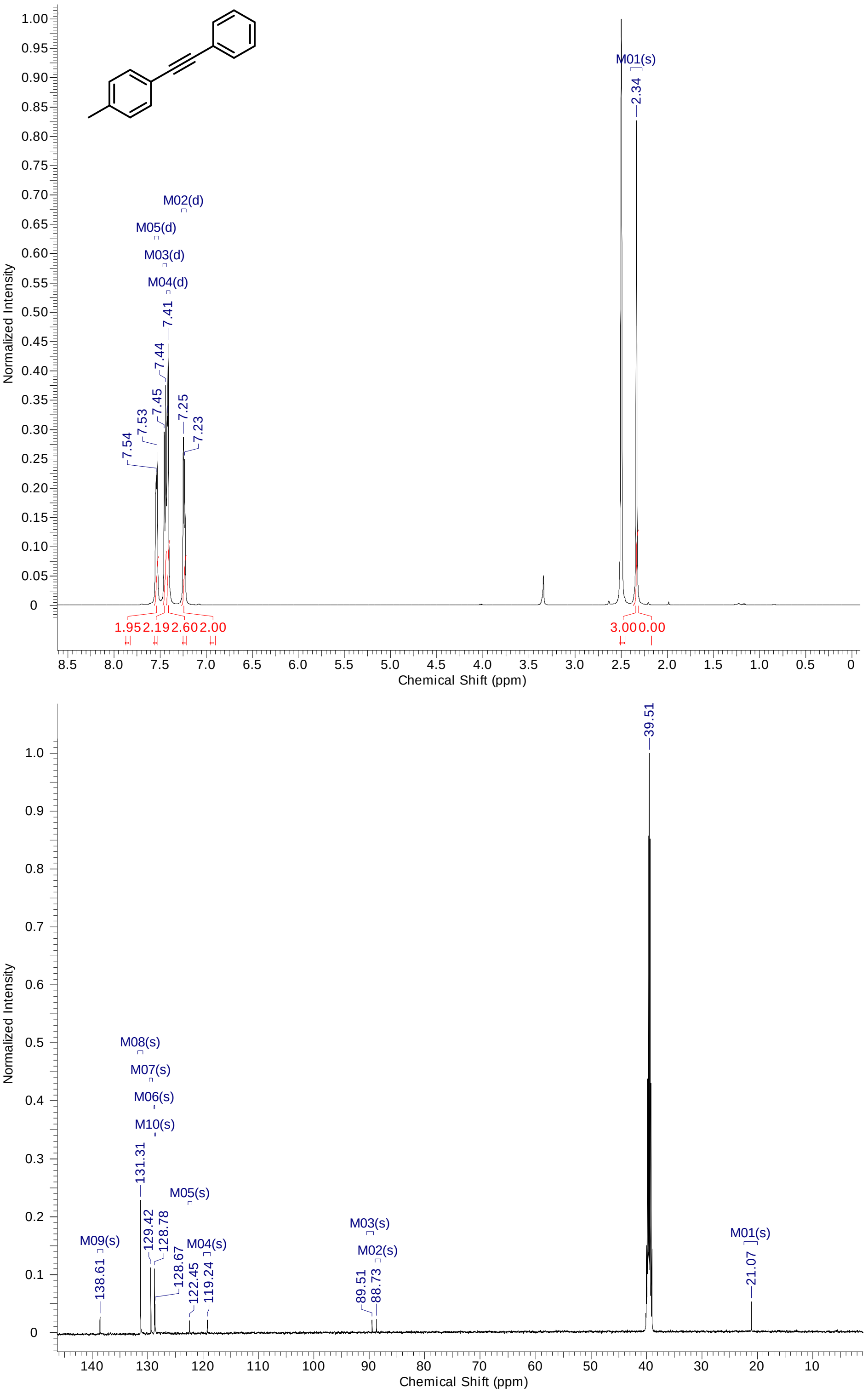

Figure S10. ^{1}H- and ^{13}C-NMR spectra of **7**.

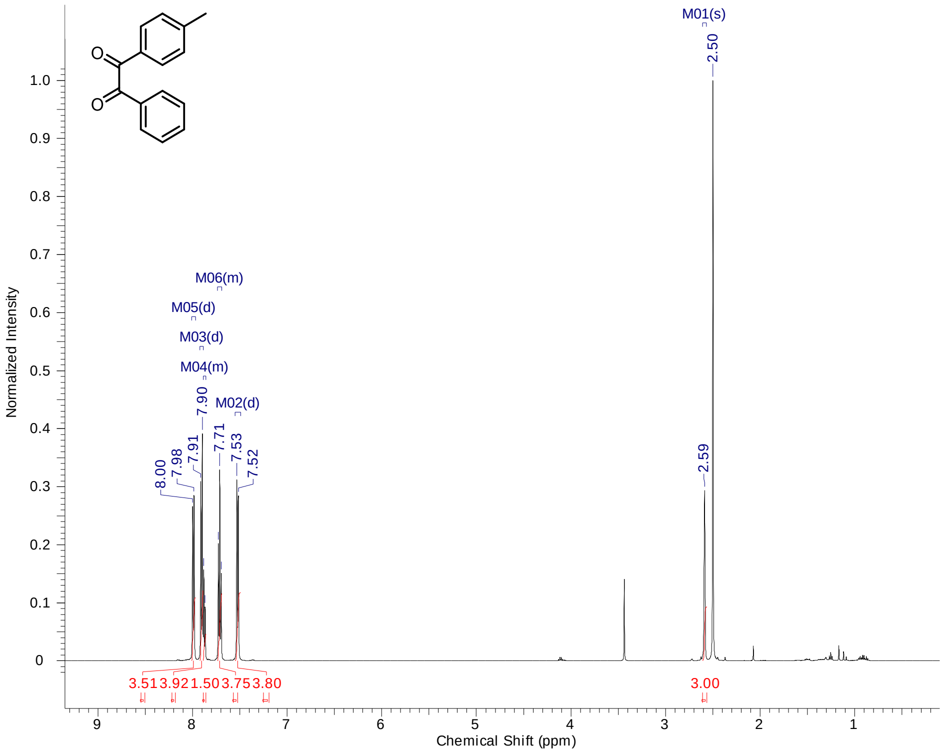

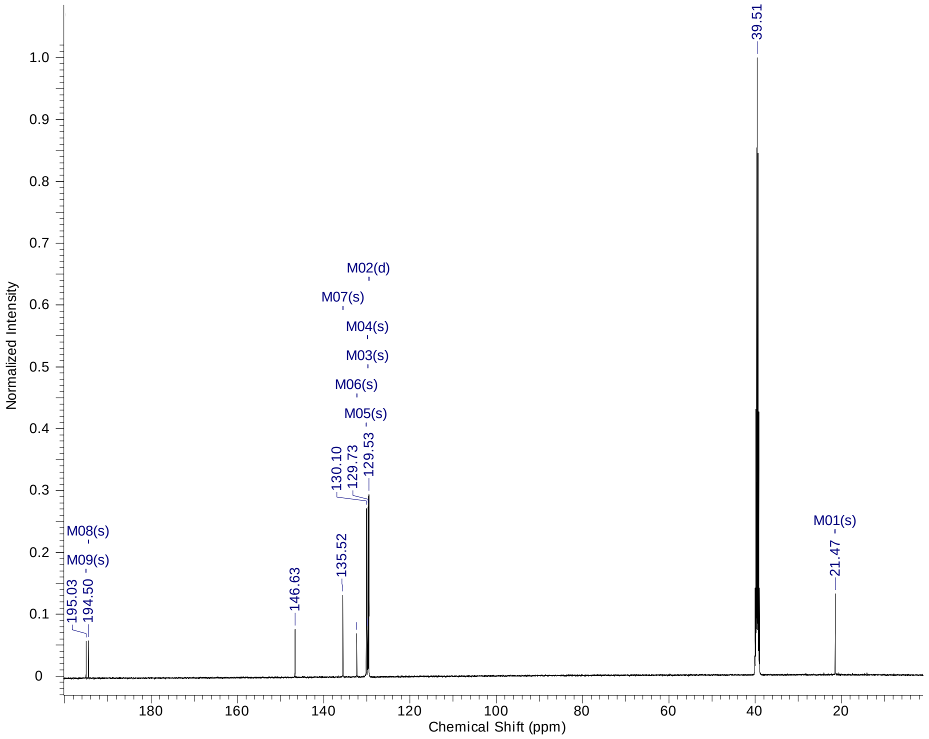

Figure S11. ^{1}H- and ^{13}C-NMR spectra of **8**.

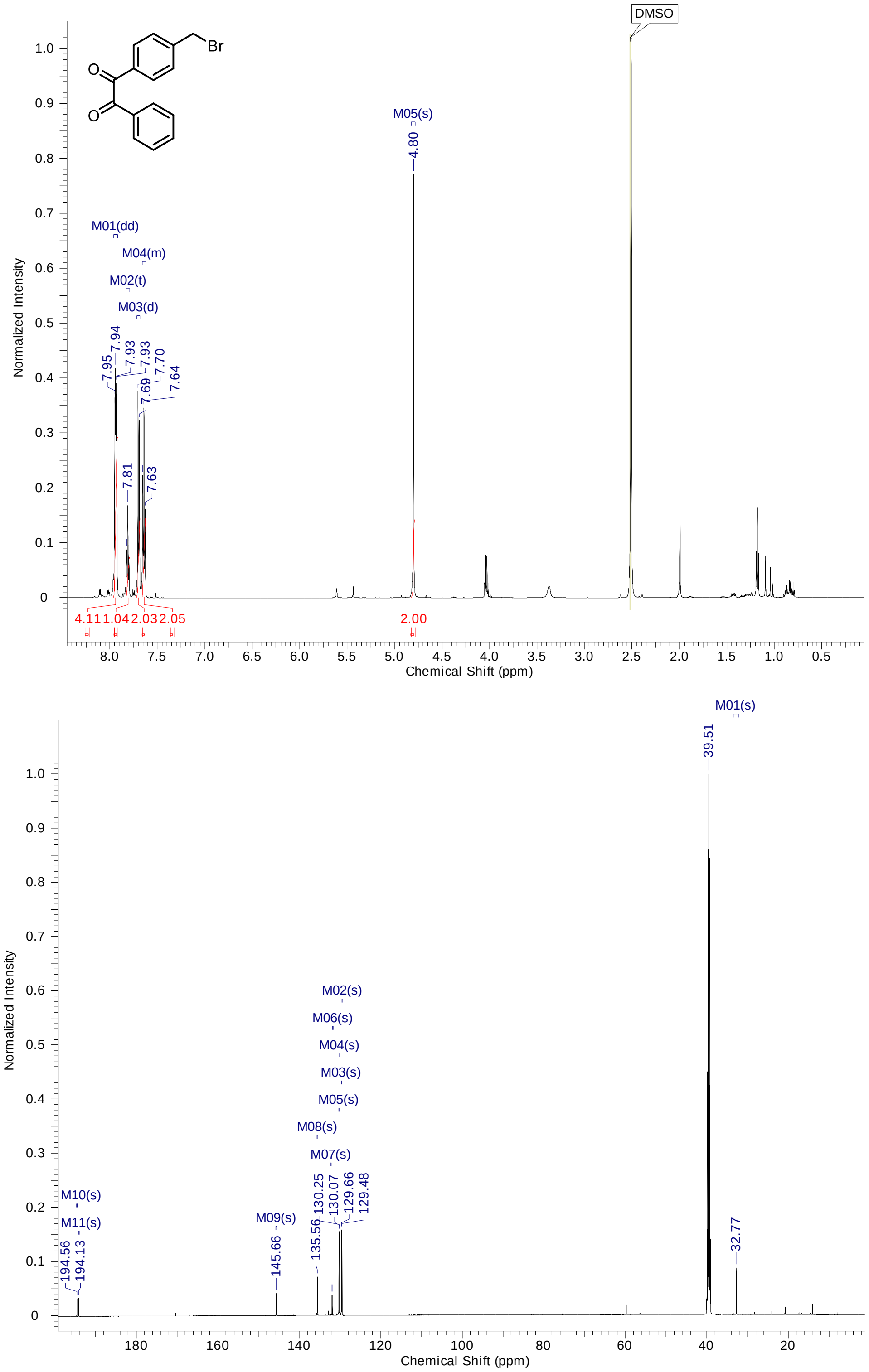

Figure S12. ^{1}H- and ^{13}C-NMR spectra of **9**.

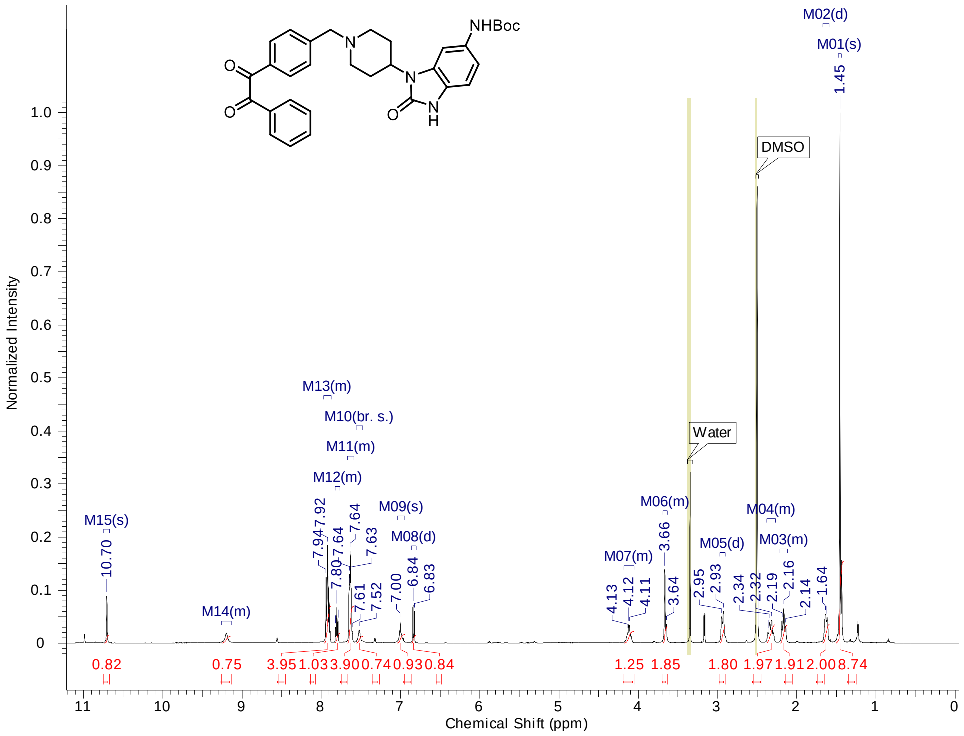

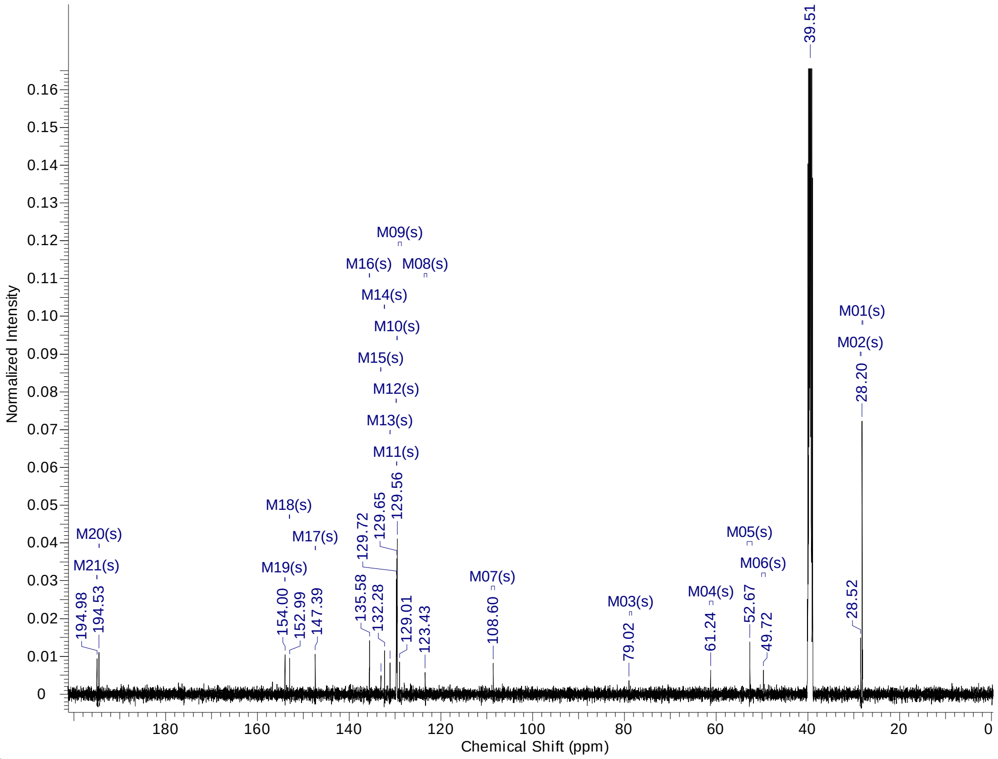

Figure S13. ^{1}H- and ^{13}C-NMR spectra of **10**.

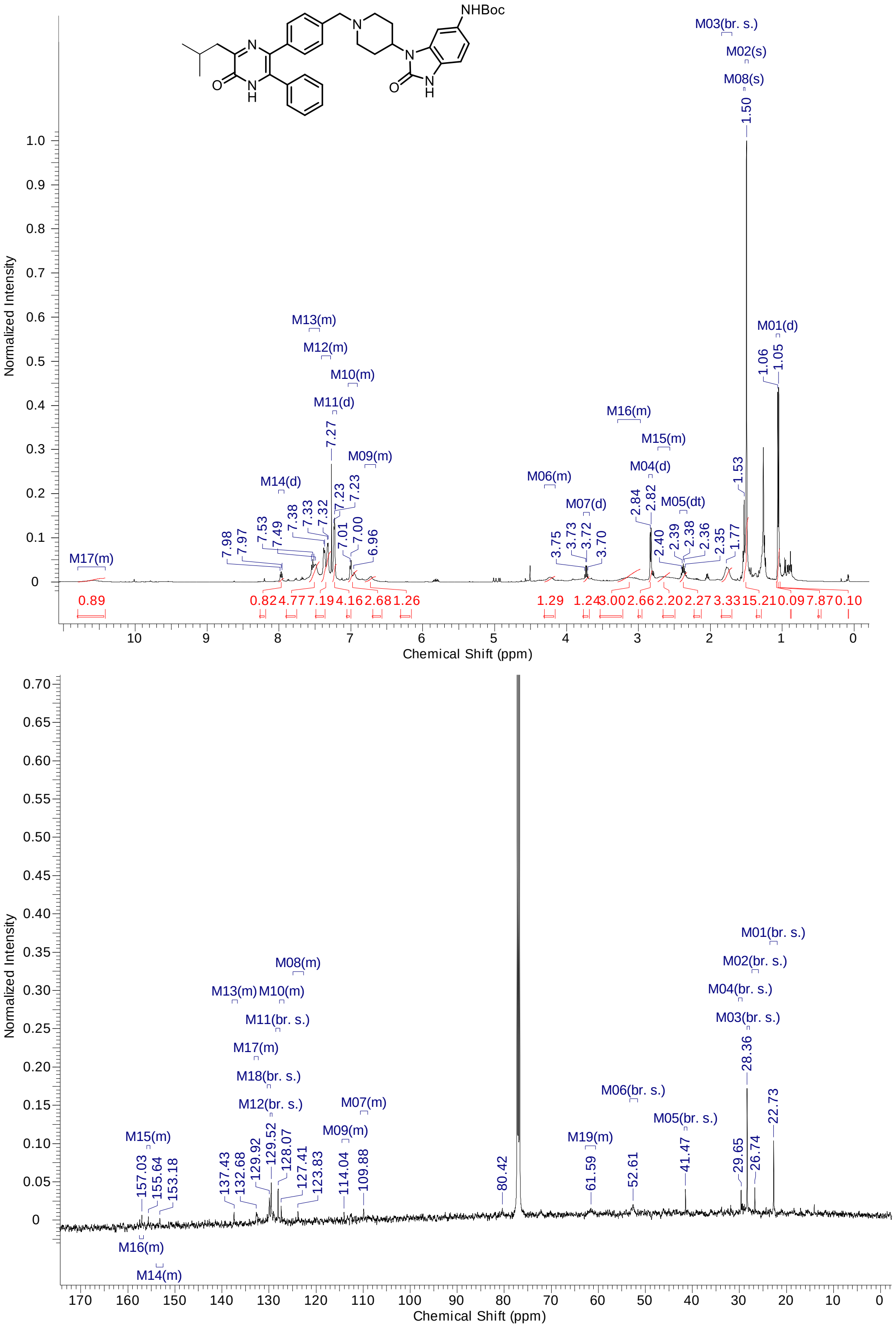

Figure S14. ^{1}H- and ^{13}C-NMR spectra of **11b**.

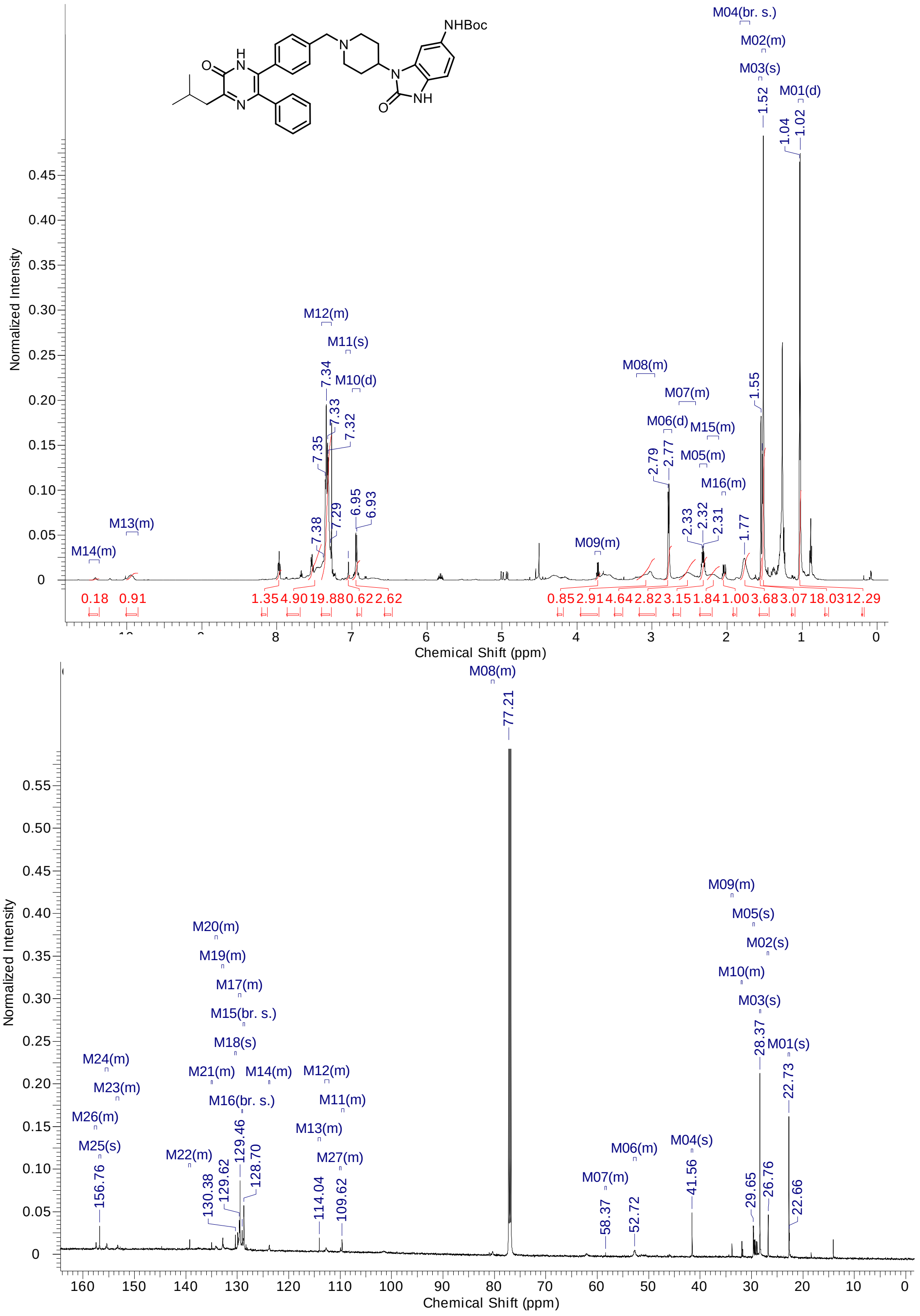

Figure S15. ^{1}H- and ^{13}C-NMR spectra of **12b**.

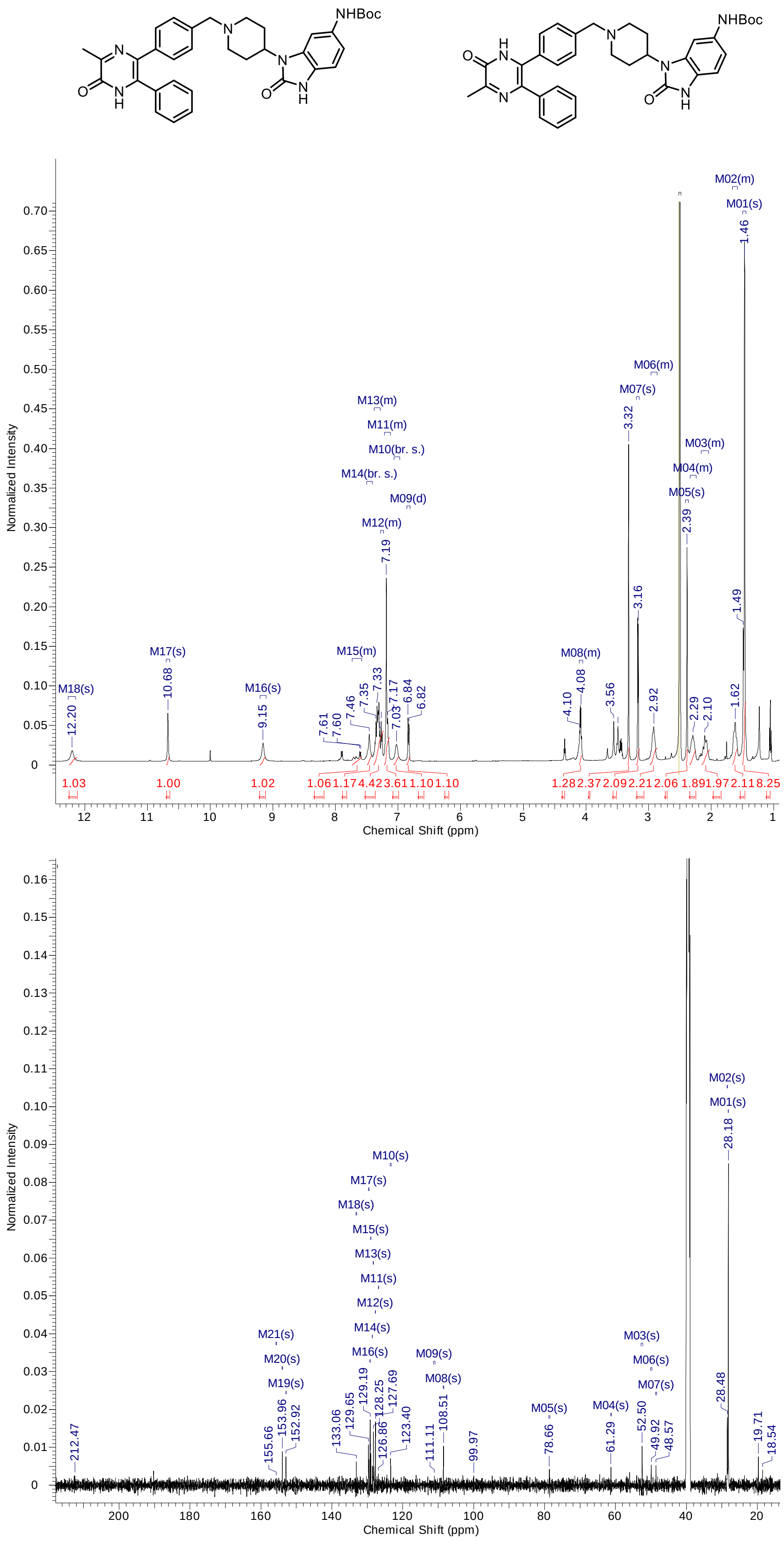

Figure S16. ^{1}H- and ^{13}C-NMR spectra of **11a** and **12a**.

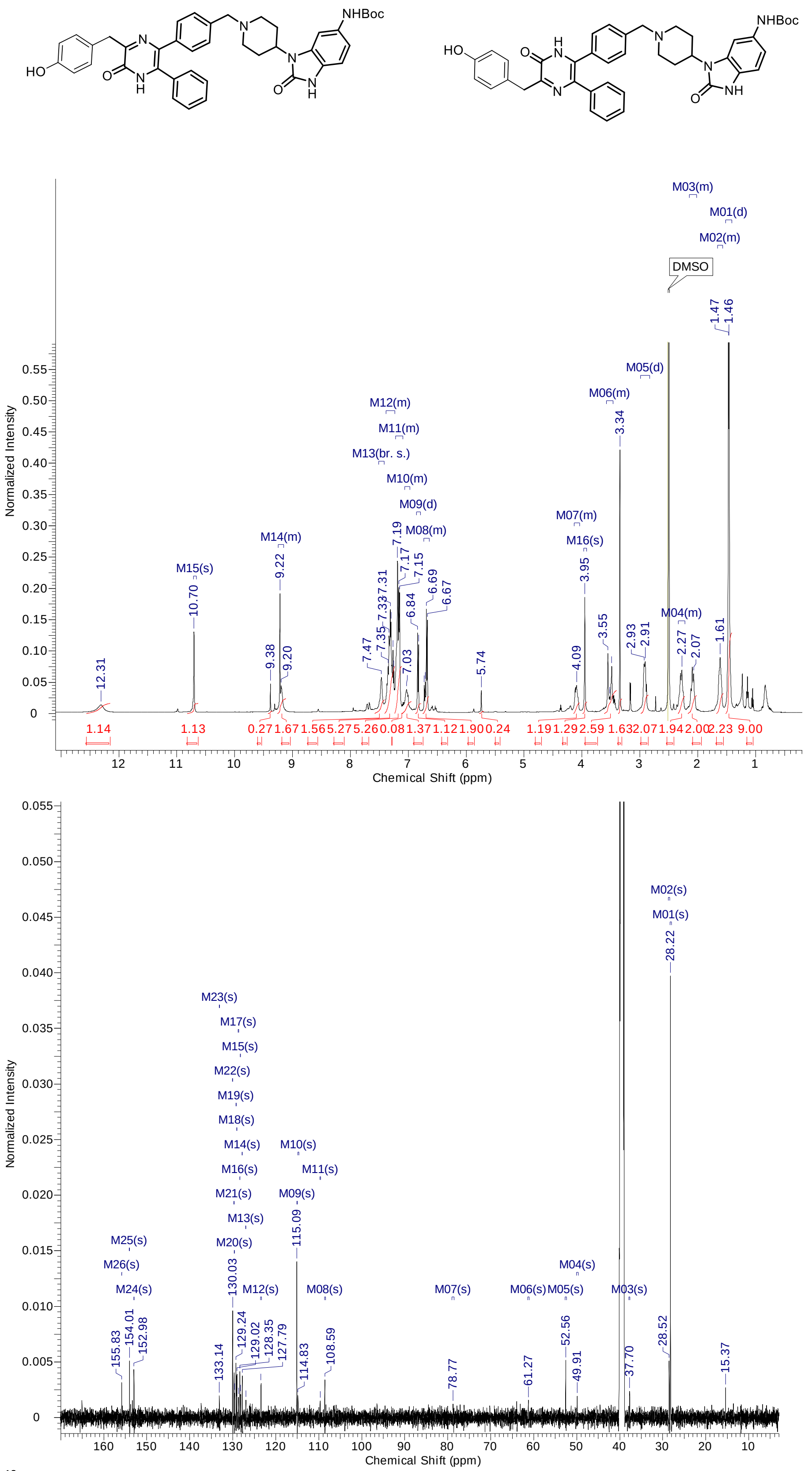

Figure S17. ^{1}H- and ^{13}C-NMR spectra of **11c** and **12c**.

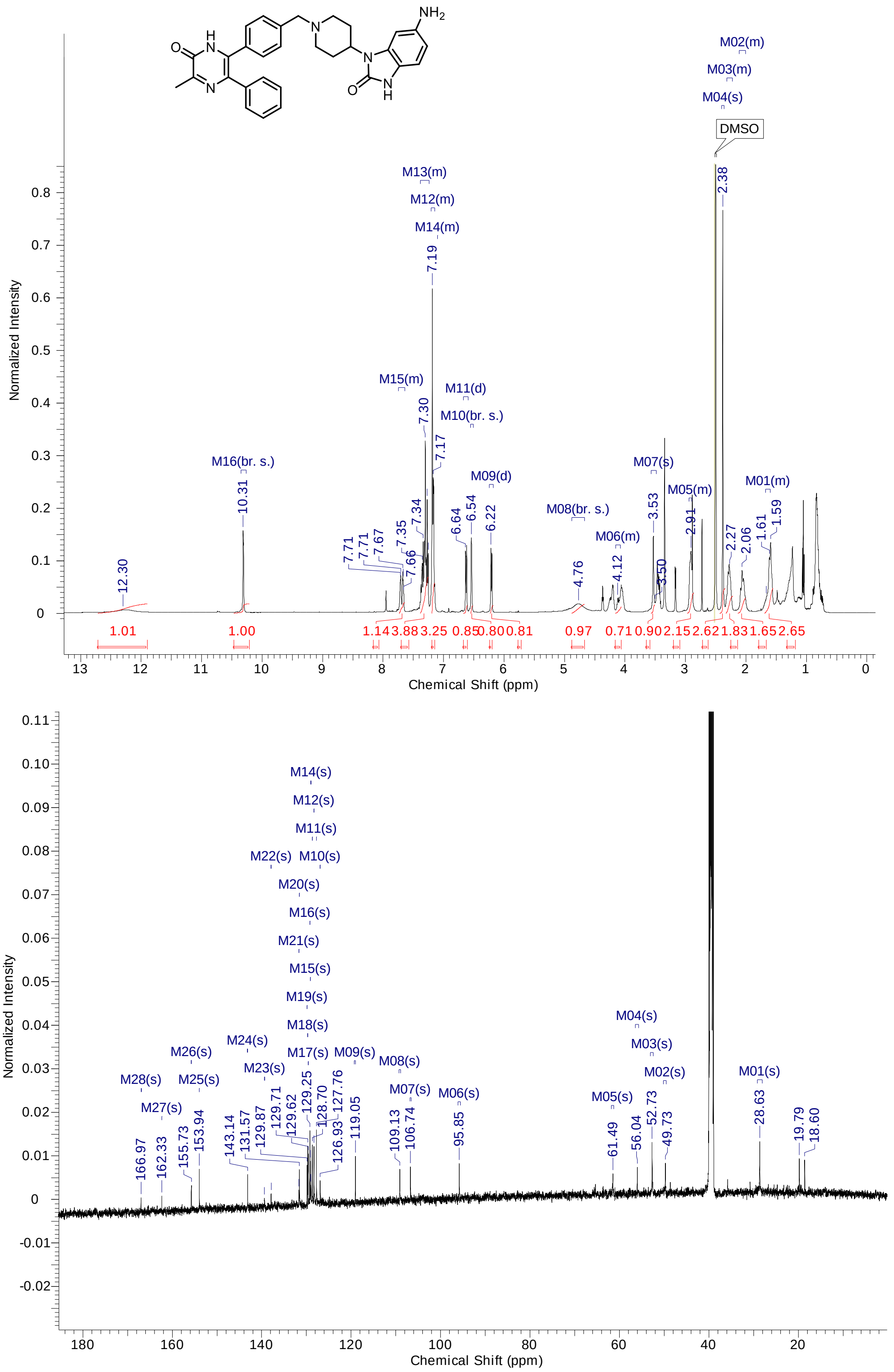

Figure S18. ^{1}H- and ^{13}C-NMR spectra of **14a**.

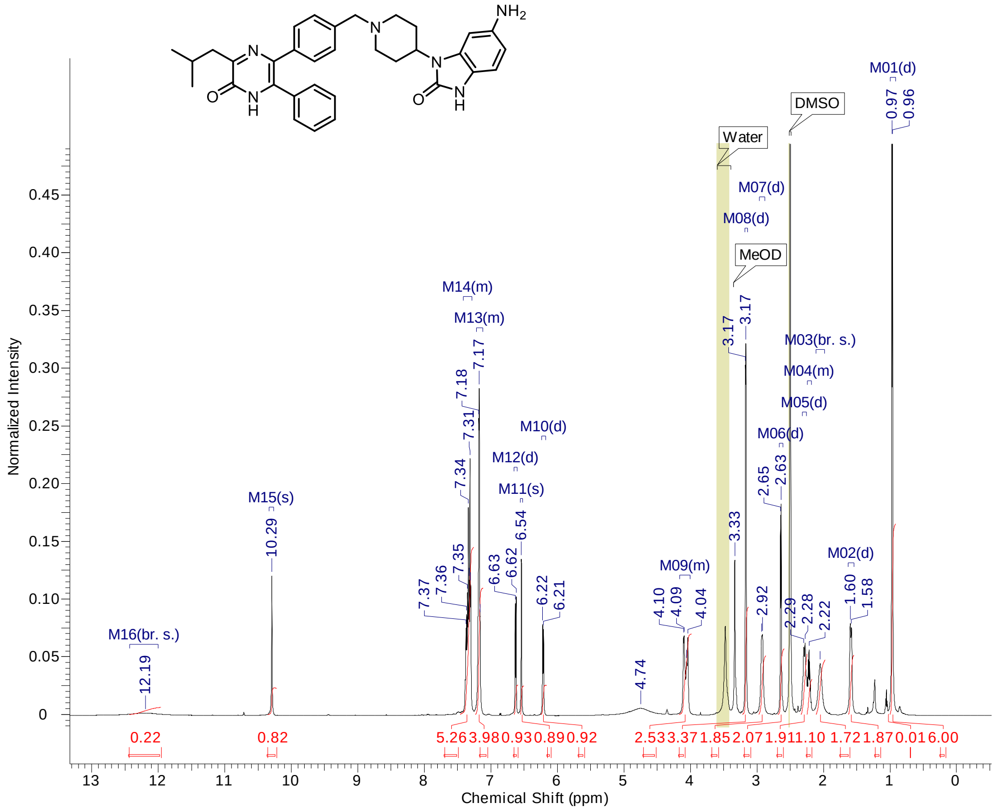

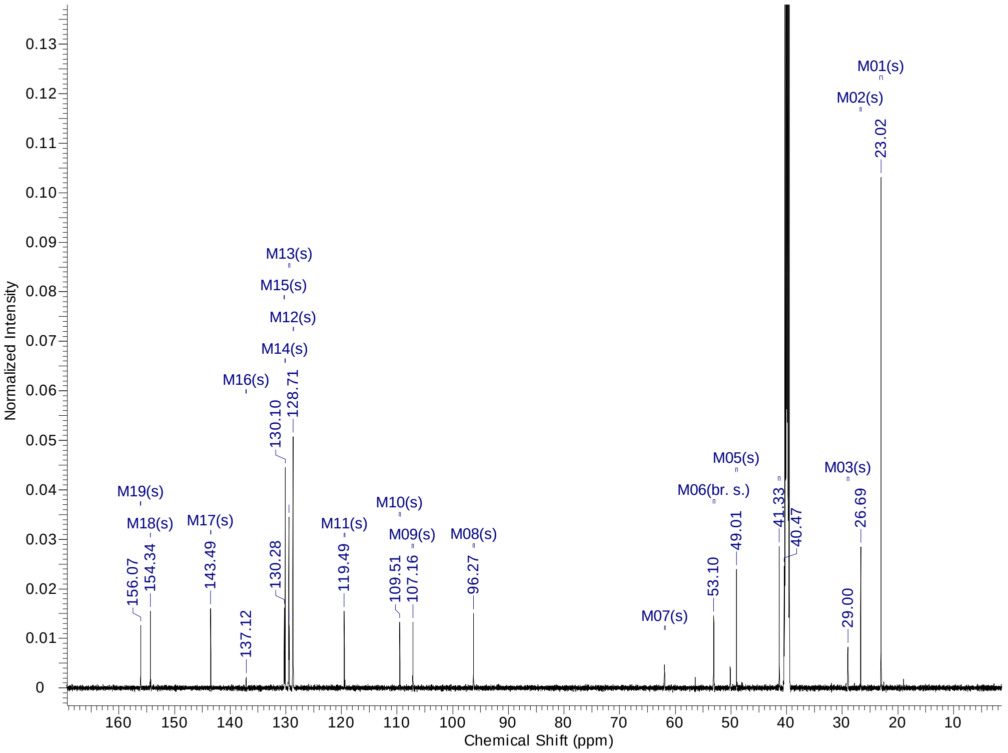

Figure S19. ^{1}H- and ^{13}C-NMR spectra of **13b.**

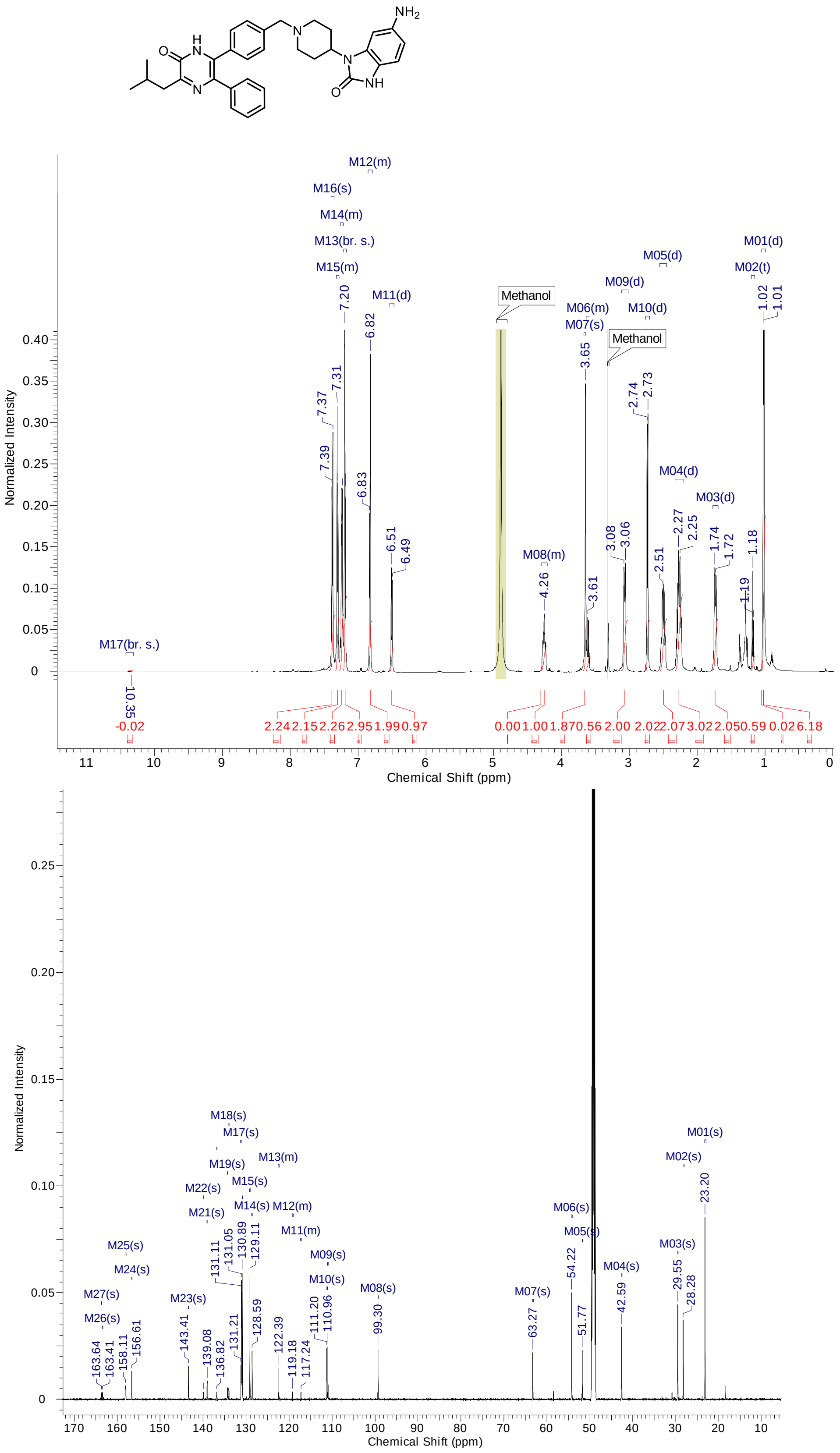

Figure S20. ^{1}H- and ^{13}C-NMR spectra of **14b.**

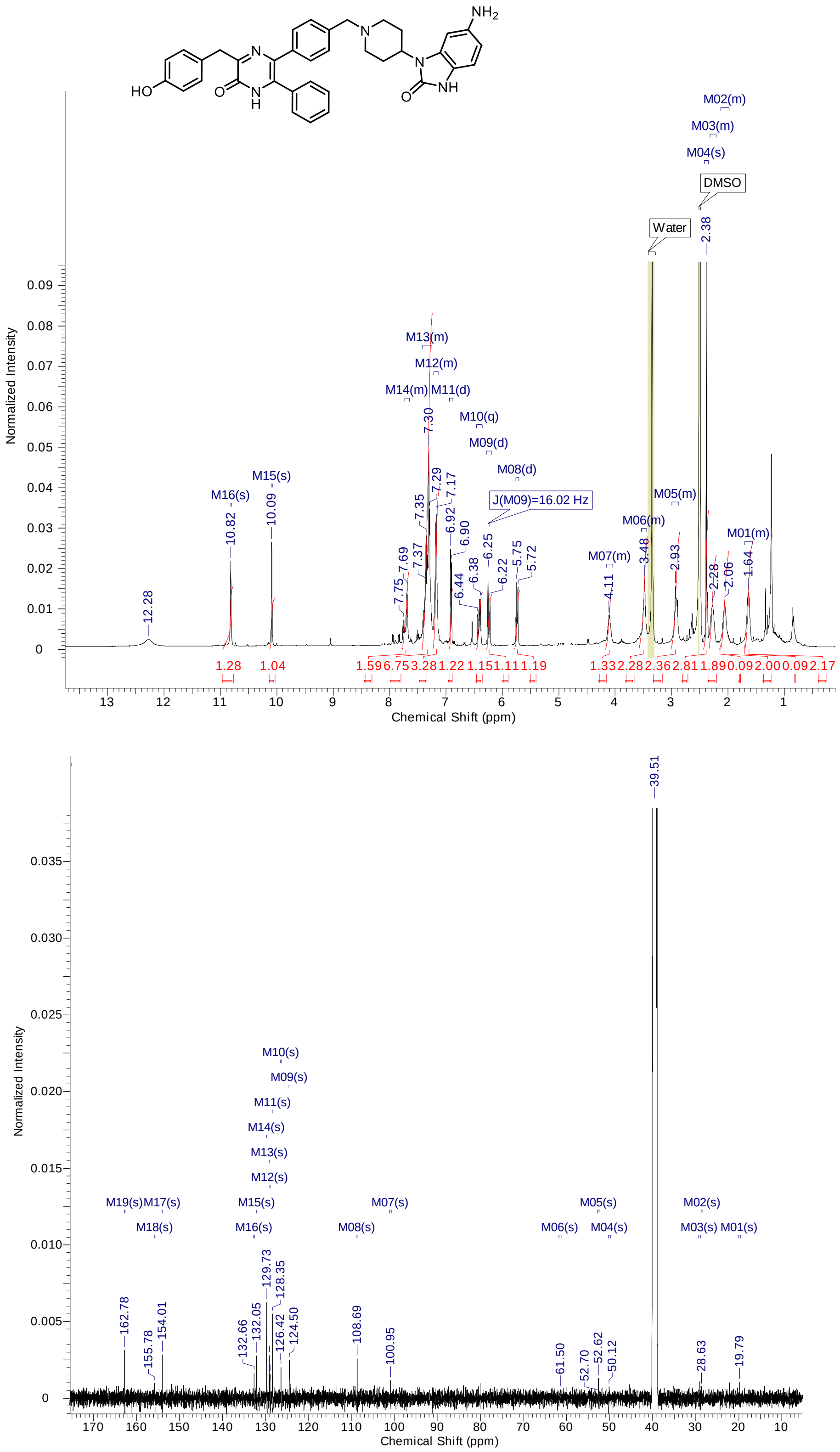

Figure S21. ^{1}H- and ^{13}C-NMR spectra of **13c.**

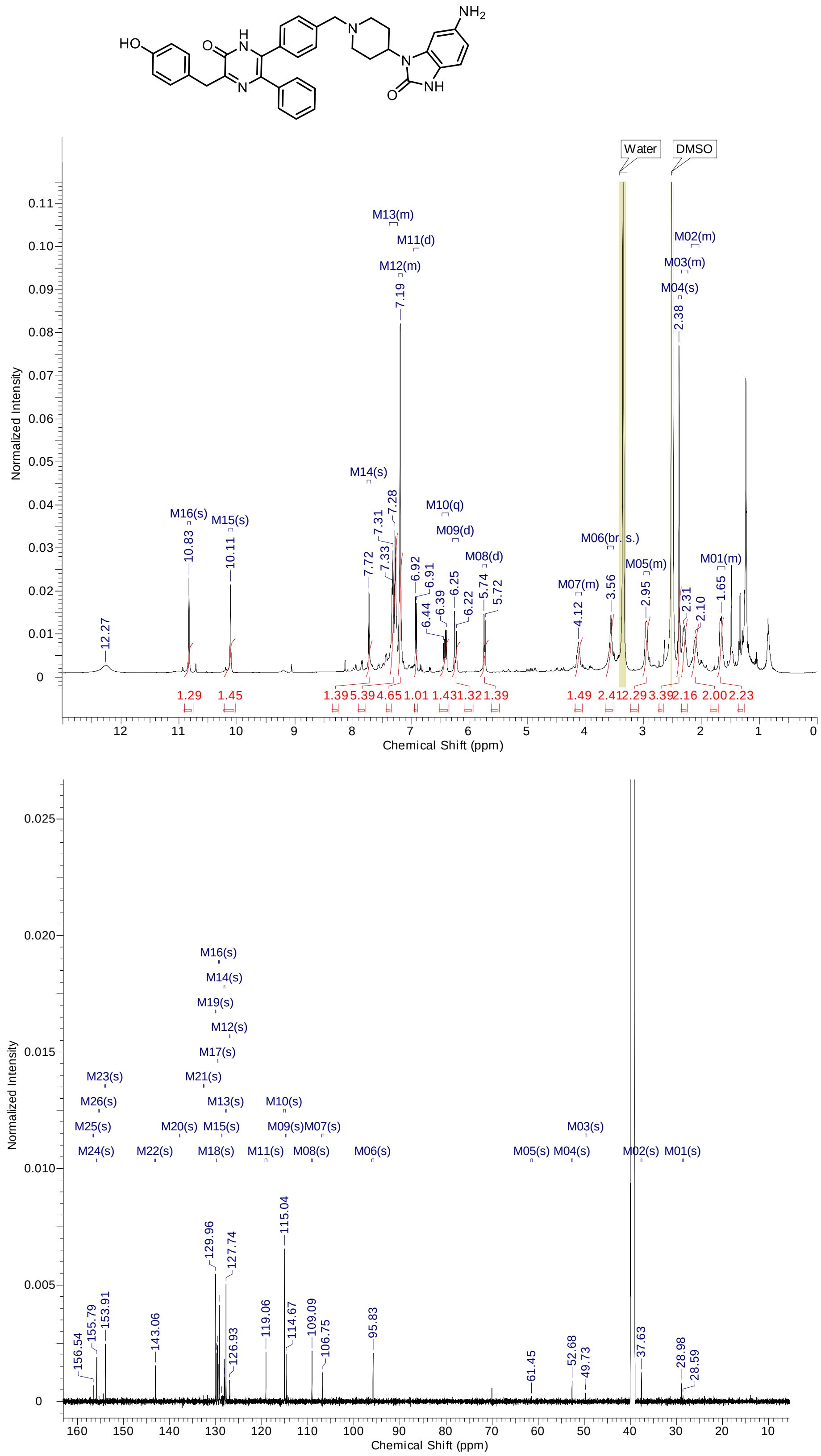

Figure S22. ^{1}H- and ^{13}C-NMR spectra of **14c.**

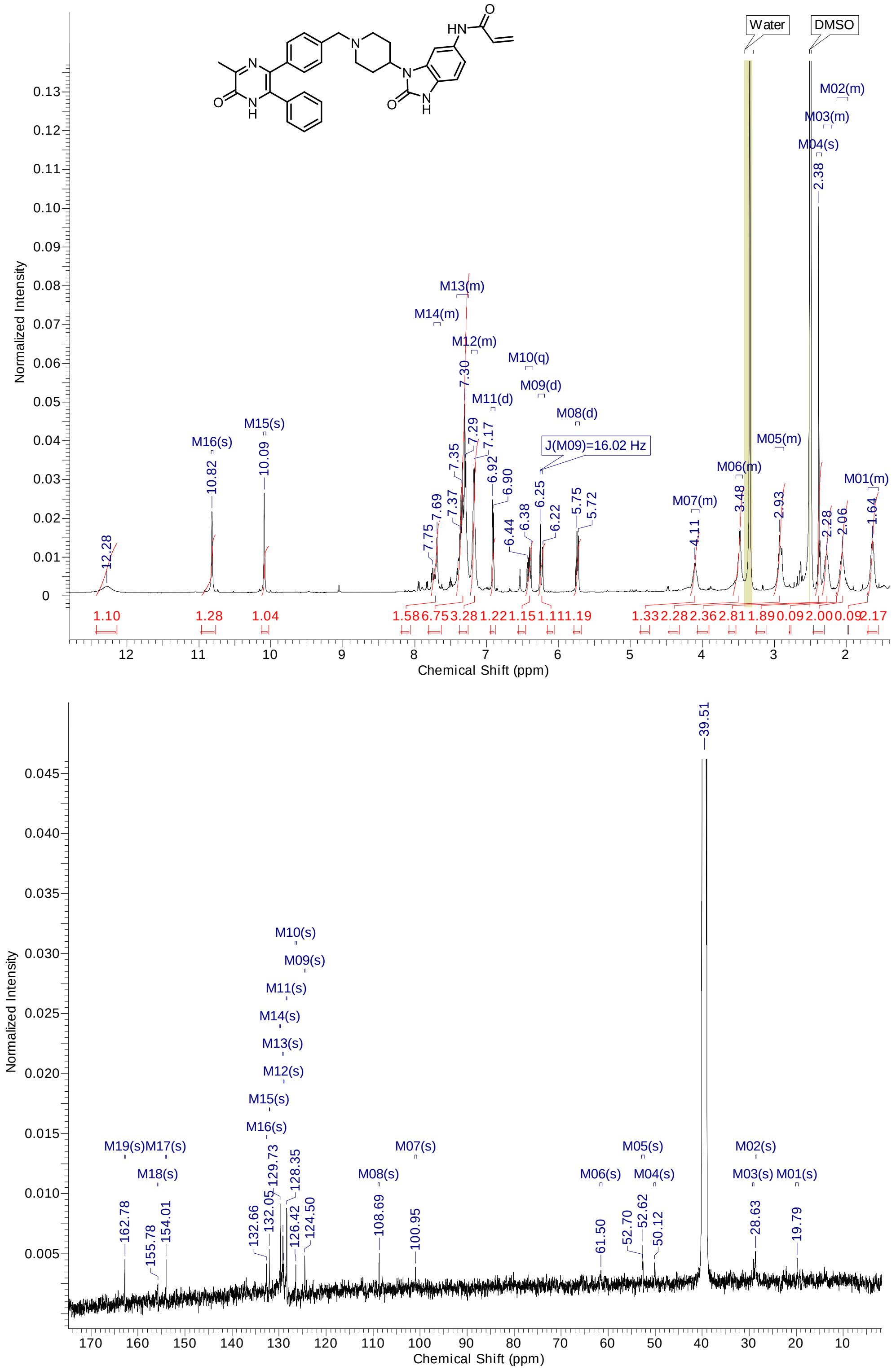

Figure S23. ^{1}H- and ^{13}C-NMR spectra of **15a.**

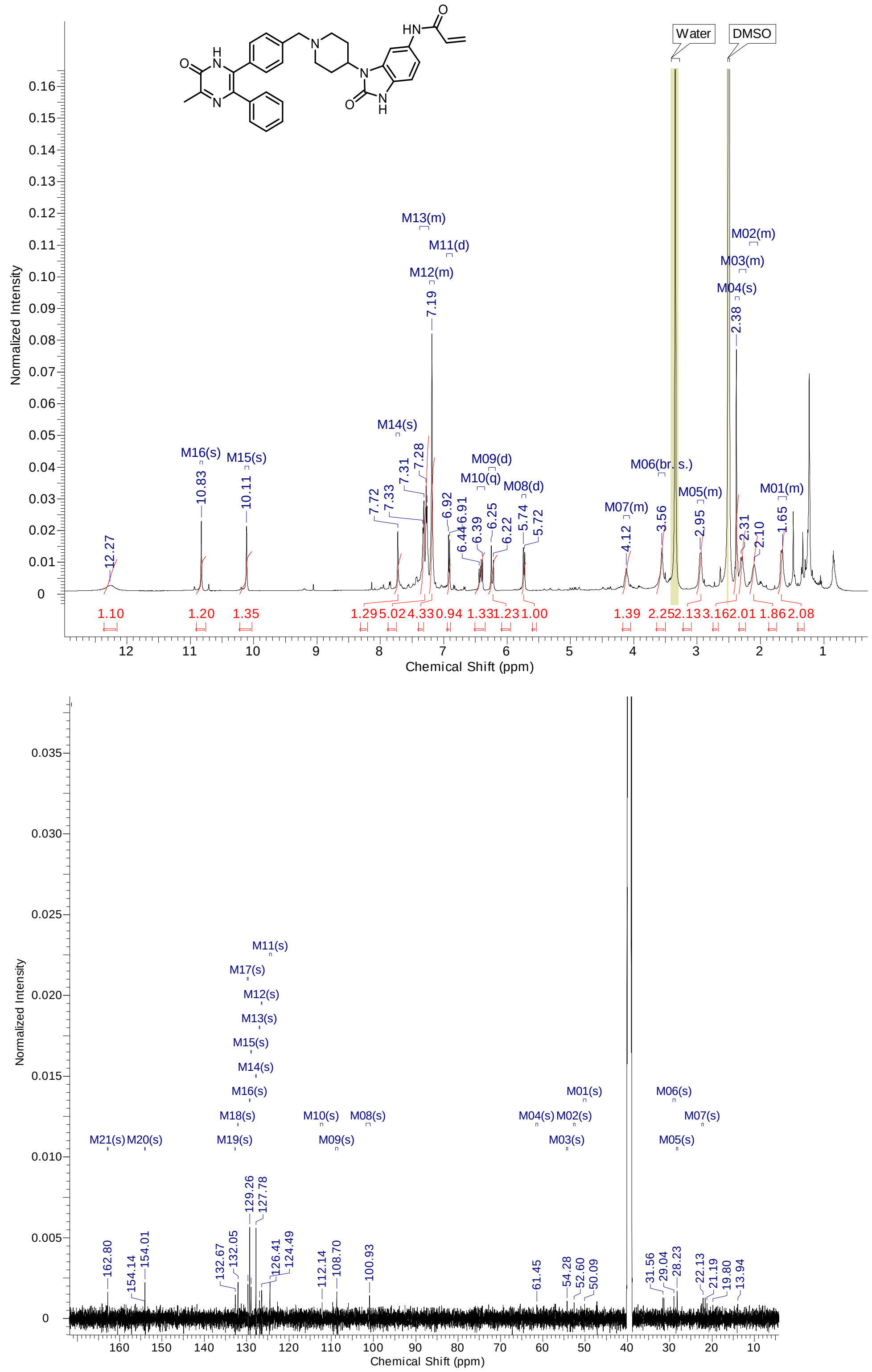

Figure S24. ^{1}H- and ^{13}C-NMR spectra of **16a.**

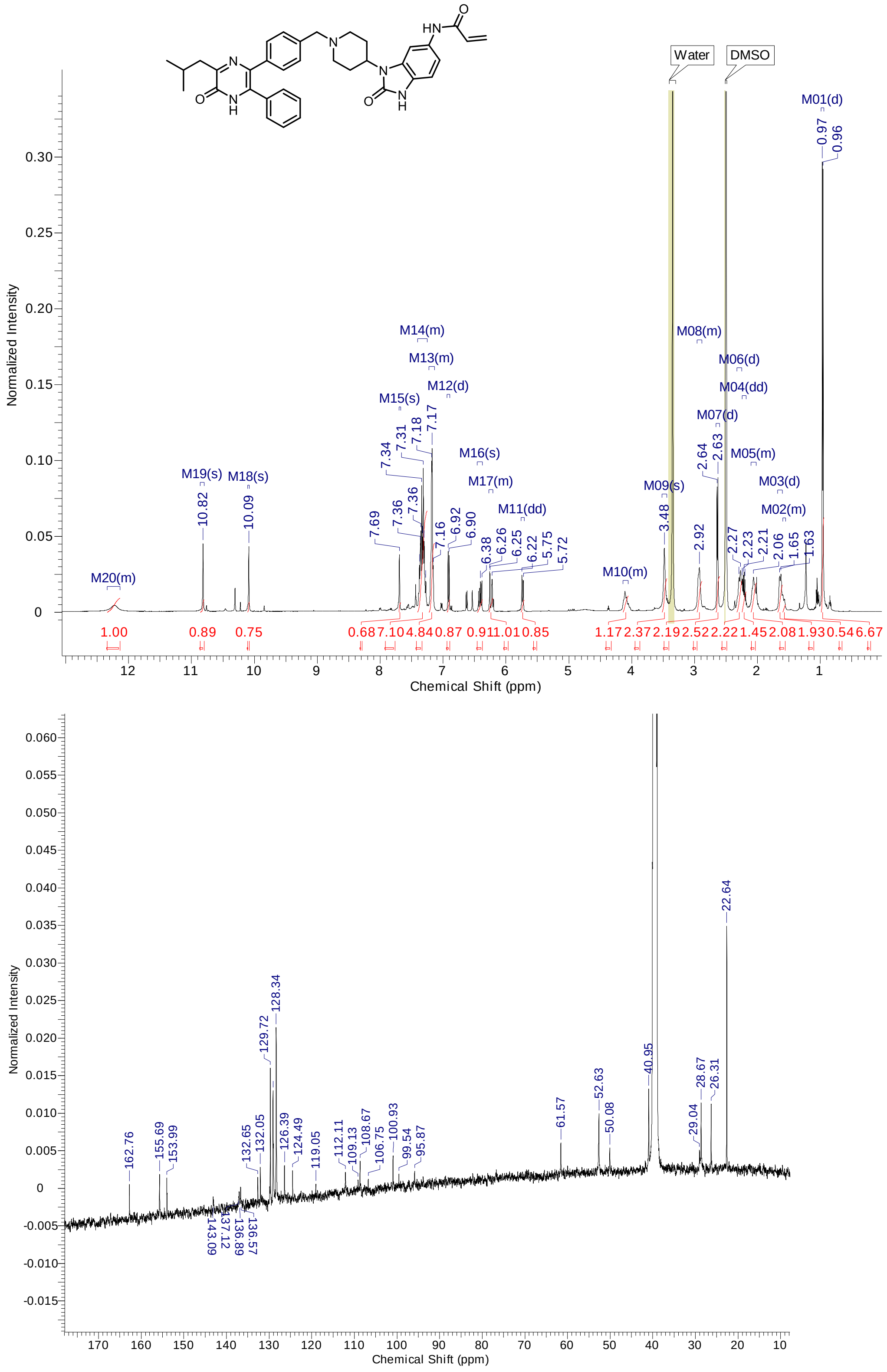

Figure S25. ^{1}H- and ^{13}C-NMR spectra of **15b.**

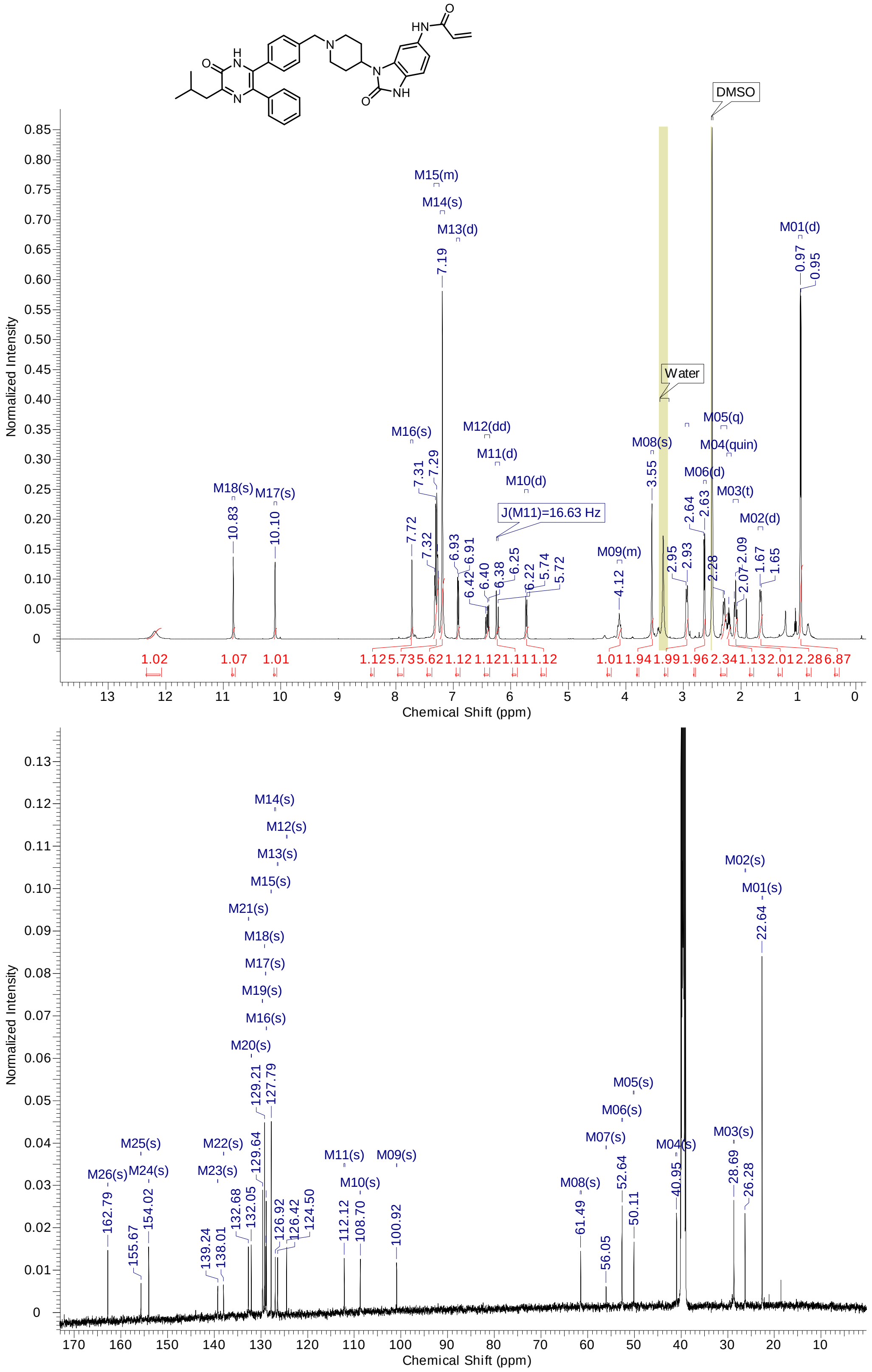

Figure S26. ^{1}H- and ^{13}C-NMR spectra of **16b.**

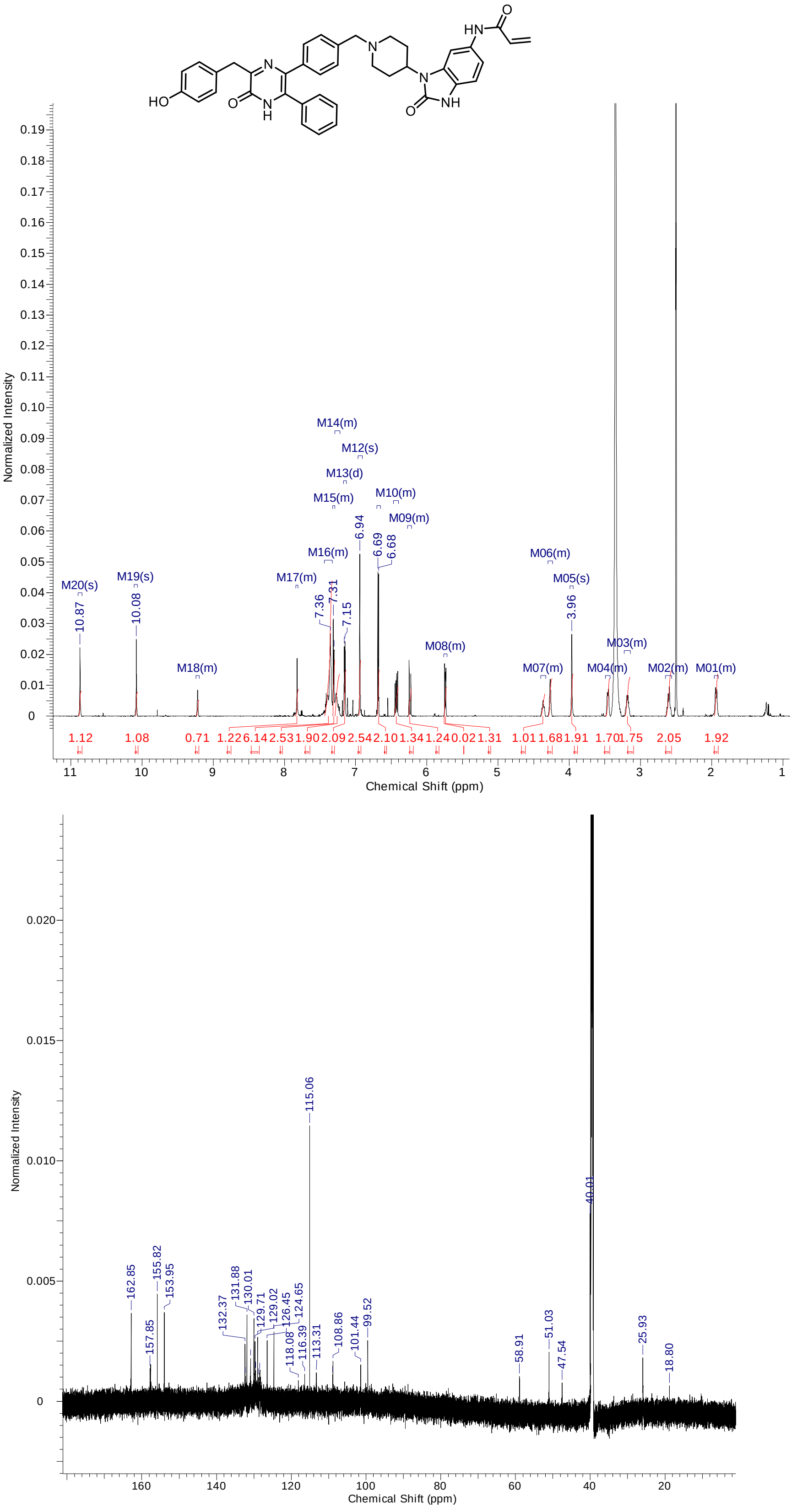

Figure S27. ^{1}H- and ^{13}C-NMR spectra of **15c.**

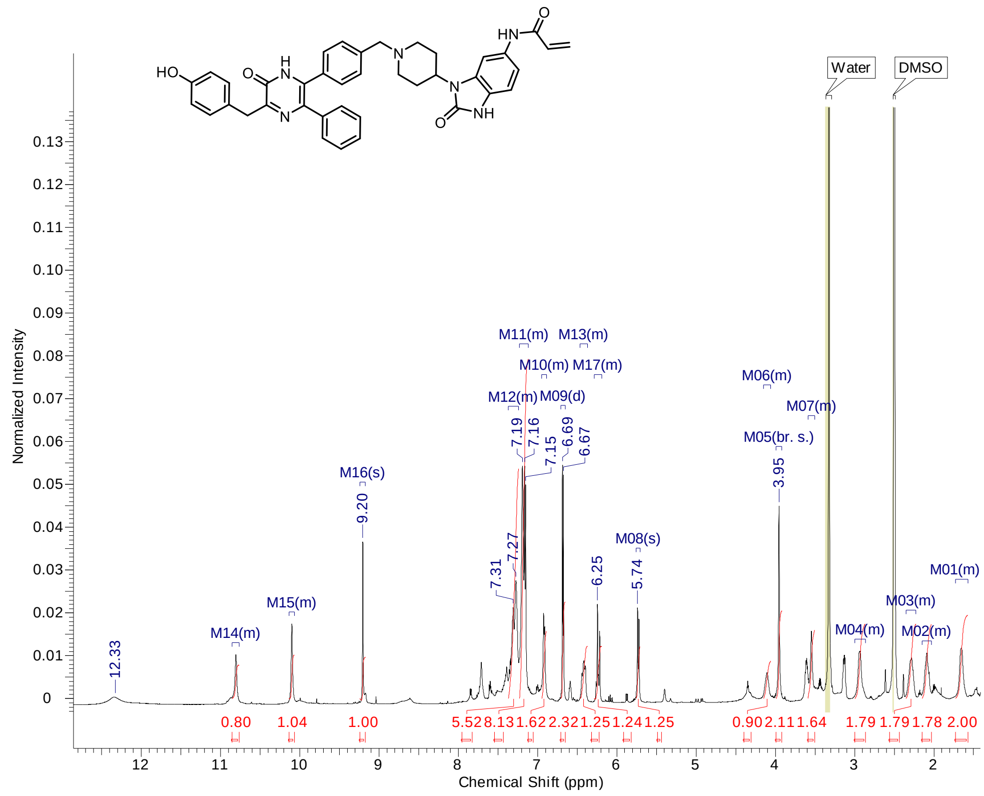

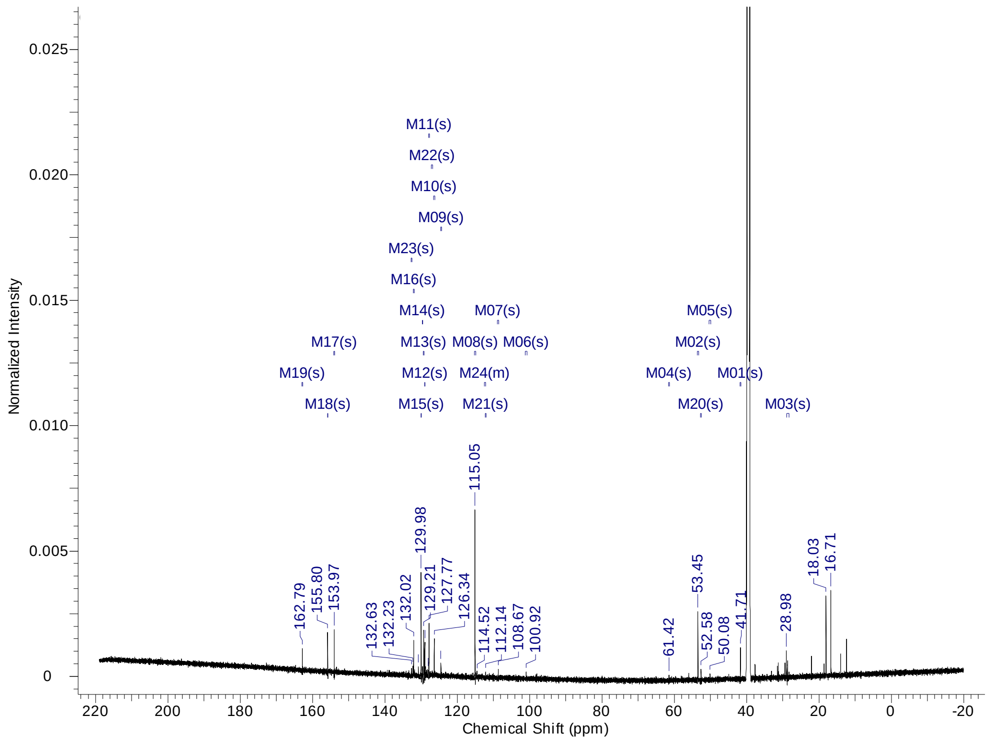

Figure S28. ^{1}H- and ^{13}C-NMR spectra of **16c.**

References

[1] F. Madeira, Y. M. Park, J. Lee, N. Buso, T. Gur, N. Madhusoodanan, P. Basutkar, A. R. N. Tivey, S. C. Potter, R. D. Finn, R. Lopez, *Nucleic Acids Res* **2019**.

[2] A. Waterhouse, C. Rempfer, F. T. Heer, G. Studer, G. Tauriello, L. Bordoli, M. Bertoni, R. Gumienny, R. Lepore, S. Bienert, T. A P. de Beer, T. Schwede, *Nucleic Acids Research* **2018**, *46*, W296-W303.

[3] Z. Fang, J. R. Simard, D. Plenker, H. D. Nguyen, T. Phan, P. Wolle, S. Baumeister, D. Rauh, *ACS chemical biology* **2015**, *10*, 279-288.

[4] B. F. Krippendorff, R. Neuhaus, P. Lienau, A. Reichel, W. Huisinga, *Journal of biomolecular screening* **2009**, *14*, 913-923.

[5] M. Strohalm, M. Hassman, B. Kosata, M. Kodicek, *Rapid communications in mass spectrometry : RCM* **2008**, *22*, 905-908.

[6] A. Shevchenko, H. Tomas, J. Havlis, J. V. Olsen, M. Mann, *Nature protocols* **2006**, *1*, 2856-2860.

[7] J. Cox, M. Mann, *Nature biotechnology* **2008**, *26*, 1367-1372.

[8] W. Kabsch, *J Appl Crystallogr* **1993**, *26*, 795-800.

[9] R. J. Read, *Acta Crystallogr D* **2001**, *57*, 1373-1382.

[10] P. Emsley, K. Cowtan, *Acta Crystallogr D* **2004**, *60*, 2126-2132.

[11] A. W. Schuttelkopf, D. M. F. van Aalten, *Acta Crystallographica Section D-Structural Biology* **2004**, *60*, 1355-1363.

[12] J. Weisner, R. Gontla, L. van der Westhuizen, S. Oeck, J. Ketzer, P. Janning, A. Richters, T. Muhlenberg, Z. Fang, A. Taher, V. Jendrossek, S. C. Pelly, S. Bauer, W. A. van Otterlo, D. Rauh, *Angewandte Chemie* **2015**, *54*, 10313-10316.

[13] P. D. Adams, P. V. Afonine, G. Bunkoczi, V. B. Chen, I. W. Davis, N. Echols, J. J. Headd, L. W. Hung, G. J. Kapral, R. W. Grosse-Kunstleve, A. J. McCoy, N. W. Moriarty, R. Oeffner, R. J. Read, D. C. Richardson, J. S. Richardson, T. C. Terwilliger, P. H. Zwart, *Acta Crystallogr D* **2010**, *66*, 213-221.

[14] V. B. Chen, W. B. Arendall, J. J. Headd, D. A. Keedy, R. M. Immormino, G. J. Kapral, L. W. Murray, J. S. Richardson, D. C. Richardson, *Acta Crystallographica Section D-Structural Biology* **2010**, *66*, 12-21.

[15] R. P. Joosten, F. Long, G. N. Murshudov, A. Perrakis, *Iucrj* **2014**, *1*, 213-220.

[16] L. Schrödinger, *The PyMOL Molecular Graphics System* **2018**, Version, 2.0.

[17] J. Weisner, I. Landel, C. Reintjes, N. Uhlenbrock, M. Trajkovic-Arsic, N. Dienstbier, J. Hardick, S. Ladigan, M. Lindemann, S. Smith, L. Quambusch, R. Scheinpflug, L. Depta, R. Gontla, A. Unger, H. Muller, M. Baumann, C. Schultz-Fademrecht, G. Gunther, A. Maghnouj, M. P. Muller, M. Pohl, C. Teschendorf, H. Wolters, R. Viebahn, A. Tannapfel, W. Uhl, J. G. Hengstler, S. A. Hahn, J. T. Siveke, D. Rauh, *Cancer research* **2019**, *79*, 2367-2378.

[18] P. Wolle, M. P. Muller, D. Rauh, *ACS chemical biology* **2018**, *13*, 496-499.

[19] a) J.-M. Lapierre, S. Eathiraj, D. Vensel, Y. Liu, C. O. Bull, S. Cornell-Kennon, S. Iimura, E. W. Kelleher, D. E. Kizer, S. Koerner, S. Makhija, A. Matsuda, M. Moussa, N. Namdev, R. E. Savage, J. Szwaya, E. Volckova, N. Westlund, H. Wu, B. Schwartz, *Journal of medicinal chemistry* **2016**, *59*, 6455-6469; b) O. Politz, F. Siegel, L. Bärfacker, U. Bömer, A. Hägebarth, W. J. Scott, M. Michels, S. Ince, R. Neuhaus, K. Meyer, A. E. Fernández-Montalván, N. Liu, F. von Nussbaum, D. Mumberg, K. Ziegelbauer, *International Journal of Cancer* **2017**, *140*, 449-459.

Author Contributions

Organic synthesis (LQ, NU), sequence alignment and homology-model (LQ), X-ray crystallography (IL, MPM), biochemical analysis (JW, LD, LQ), cellular evaluation (IL, LD, FG, KA, JW), data analysis (all authors), drafting the manuscript (LQ, MPM, DR) and project administration (JTS, DR).
